# Consuming viscous prey: a novel protein-secreting delivery system in neotropical snail-eating snakes

**DOI:** 10.1186/1471-2148-14-58

**Published:** 2014-03-25

**Authors:** Hussam Zaher, Leonardo de Oliveira, Felipe G Grazziotin, Michelle Campagner, Carlos Jared, Marta M Antoniazzi, Ana L Prudente

**Affiliations:** 1 Museu de Zoologia da Universidade de São Paulo, Avenida Nazaré 481, São Paulo, SP CEP 04263-000, Brazil; 2 Programa de Pós Graduação em Zoologia, Universidade Estadual Paulista, Avenida 24A 1515, Rio Claro, SP CEP 13506-900, Brazil; 3 Museu Biológico, Instituto Butantan, Avenida Vital Brazil 1500, São Paulo, SP CEP 05503-900, Brazil; 4 Laboratório de Biologia Celular, Instituto Butantan, Avenida Vital Brazil 1500, São Paulo, SP CEP 05503-900, Brazil; 5 Museu Paraense Emílio Goeldi, Avenida Magalhães Barata 376, Belém, PA CEP 66040-170, Brazil

**Keywords:** Phylogeny, Evolution, Dipsadinae, Glands, Secretion, Muscles, Goo-eaters

## Abstract

**Background:**

Efficient venom delivery systems are known to occur only in varanoid lizards and advanced colubroidean snakes among squamate reptiles. Although components of these venomous systems might have been present in a common ancestor, the two lineages independently evolved strikingly different venom gland systems. In snakes, venom is produced exclusively by serous glands in the upper jaw. Within the colubroidean radiation, lower jaw seromucous infralabial glands are known only in two distinct lineages–the basal pareatids and the more advanced Neotropical dipsadines known as “goo-eating snakes”. Goo-eaters are a highly diversified, ecologically specialized clade that feeds exclusively on invertebrates (e.g., gastropod molluscs and annelids). Their evolutionary success has been attributed to their peculiar feeding strategies, which remain surprisingly poorly understood. More specifically, it has long been thought that the more derived Dipsadini genera *Dipsas* and *Sibynomorphus* use glandular toxins secreted by their infralabial glands to extract snails from their shells.

**Results:**

Here, we report the presence in the tribe Dipsadini of a novel lower jaw protein-secreting delivery system effected by a gland that is not functionally related to adjacent teeth, but rather opens loosely on the oral epithelium near the tip of the mandible, suggesting that its secretion is not injected into the prey as a form of envenomation but rather helps control the mucus and assists in the ingestion of their highly viscous preys. A similar protein-secreting system is also present in the goo-eating genus *Geophis* and may share the same adaptive purpose as that hypothesized for Dipsadini. Our phylogenetic hypothesis suggests that the acquisition of a seromucous infralabial gland represents a uniquely derived trait of the goo-eating clade that evolved independently twice within the group as a functionally complex protein-secreting delivery system.

**Conclusions:**

The acquisition by snail-eating snakes of such a complex protein-secreting system suggests that the secretion from the hypertrophied infralabial glands of goo-eating snakes may have a fundamental role in mucus control and prey transport rather than envenomation of prey. Evolution of a functional secretory system that combines a solution for mucus control and transport of viscous preys is here thought to underlie the successful radiation of goo-eating snakes.

## Background

The origin and evolution of the venom-delivery system in snakes has been a subject of considerable debate [1-7]. Recent contributions were successful in providing new, stimulating insights on the long-standing problem of the origin of the ophidian upper jaw venom system [6,7]. However, much remains to be elucidated regarding the function and morphological diversification of venomous systems in snakes [8-11]. Among advanced colubroids [12] the dipsadine “goo-eating” snakes are known to possess a peculiar lower jaw seromucous secreting system [10,13-17] that may be paralleled only in pareatids, a basal lineage of snail-eating colubroideans that also seems to possess a similar (but not homologous) lower jaw seromucous system [8,15,16].

“Goo-eaters” were originally defined as a clade of eight Neotropical genera belonging to the Subfamily Dipsadinae [18]. Among these, *Adelphicos*, *Atractus*, and *Geophis* are known to feed mainly on earthworms, whereas *Ninia*, *Dipsas*, *Sibynomorphus*, *Sibon*, and *Tropidodipsas* are molluscivorous specialists that feed mainly on slugs and snails (see Additional file 1). The latter four genera are often called “snail-eating” snakes [19]. *Chersodromus* and three recently described genera–*Chapinophis*, *Omoadiphas*, and *Plesiodipsas–*also seem to belong to the goo-eating dipsadine snake assemblage [20-27]. However, virtually nothing is known about their relationships and feeding strategies due to their cryptic habits and scarcity in collections.

Our investigation of the cephalic glandular and muscular systems of snail-eating snakes revealed a novel lower jaw protein-secretion delivery system that we describe and compare with other goo-eater genera. The new system reported here represents the first protein-secreting apparatus in snakes that is not functionally related to a specialized tooth or tooth row, but rather opens loosely on the epithelium of the mouth floor. The acquisition by the derived snail-eating snakes of such a complex protein-secreting system suggests that the secretion from the hypertrophied infralabial glands of goo-eating snakes may have a fundamental role in mucus control and prey transport rather than envenomation of prey [17]. Evolution of a functional secretory system that combines a solution for mucus control and transport of viscous prey is here thought to underlie the successful radiation of goo-eating snakes, a group that includes three of the most speciose genera of advanced snakes known so far.

## Results

### The infralabial glands and the epithelium of the floor of the mouth of Dipsadini

In order to investigate the anatomical specializations of the protein-secretion delivery system associated to the lower jaw of snail-eating snakes, we analyzed representatives of four of the five known genera of Dipsadini. We also dissected representatives of five other goo-eating genera and 29 additional genera of Dipsadinae (see Additional file 2 and Methods).

**Figure 1:**
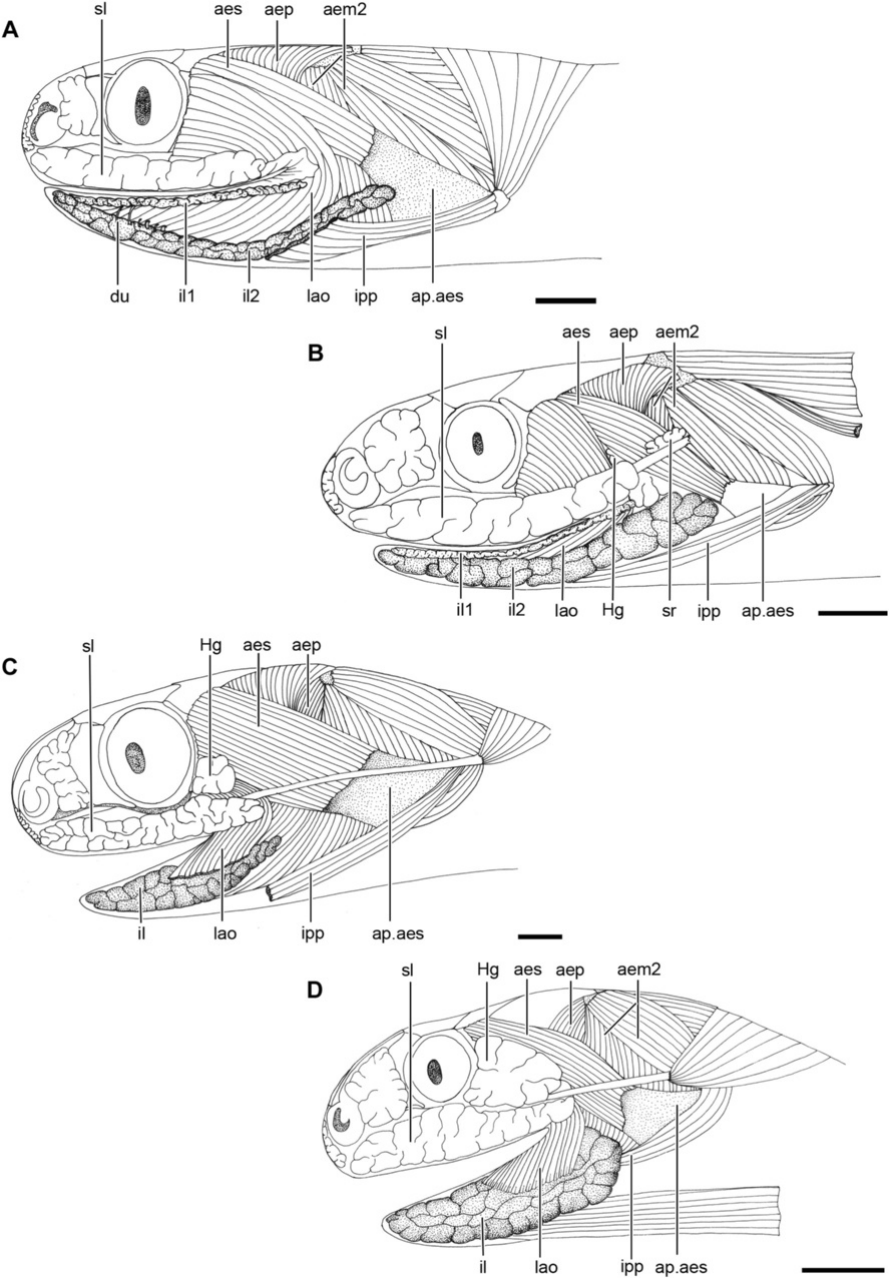
**Head muscles and glands of Dipsadini.** Lateral view of the head of *Dipsas neivai* (IBSP 54935) **(A)**, *Sibynomorphus ventrimaculatus* (IBSP 17228) **(B)**, *Sibon nebulatus* (KU 112474) **(C)**, and *Tropidodipsas sartorii* (KU 157636) **(D)**, showing the location of the infralabial gland (il) with respect to head muscles and mandible. Abbreviations: aem2, muscle *adductor mandibulae externus medialis pars posterior*; aep, muscle *adductor mandibulae externus profundus*; aes, muscle *adductor mandibulae externus superficialis*; ap.aes, aponeurose of muscle *adductor mandibulae externus superficialis*; du, ducts; Hg, harderian gland; il1, lateral, mucous infralabial gland; il2, ventrolateral, seromucous infralabial gland; ipp, muscle *intermandibularis posterior pars posterior*; lao, muscle *levator anguli oris*; sl, supralabial gland; sr, superior rictal gland. Scale bar in all pictures = 2.5 mm.

**Figure 2:**
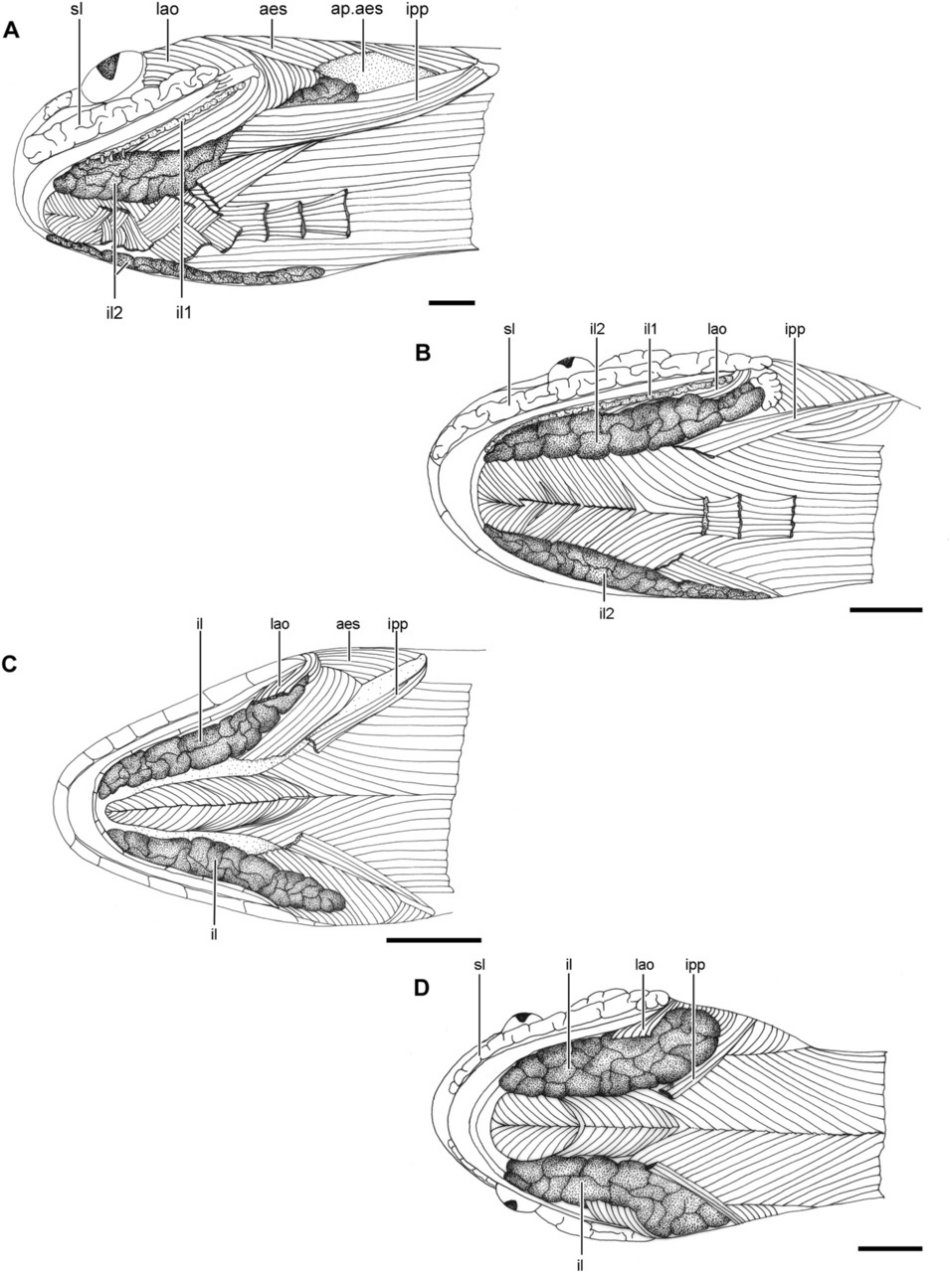
**Head muscles and glands of Dipsadini.** Ventral view of the head of *Dipsas neivai* (IBSP 54935) **(A)**, *Sibynomorphus ventrimaculatus* (IBSP 17228) **(B)**, *Sibon nebulatus* (AMNH 97068) **(C)**, and *Tropidodipsas sartorii* (KU 157636) **(D)**, showing the location of the infralabial gland (il) with respect to head muscles and mandible. Abbreviations: aes, muscle *adductor mandibulae externus superficialis*; ap.aes, aponeurose of muscle *adductor mandibulae externus superficialis*; il1, lateral, mucous infralabial gland; il2, ventrolateral, seromucous infralabial gland; ipp, muscle *intermandibularis posterior pars posterior*; lao, muscle *levator anguli oris*; sl, supralabial gland. Scale bar in all pictures = 2.5 mm.

All four available genera of Dipsadini present hypertrophied infralabial glands, a salient characteristic that distinguishes them from the remaining Dipsadinae. Besides being hypertrophied, the infralabial gland shows two distinct patterns within Dipsadini, as follows: in *Sibon* and *Tropidodipsas* (and in the goo-eaters *Atractus*, *Adelphicos*, and *Ninia*) the infralabial gland is single and located in the ventrolateral surface of the head, below the infralabial scales, whereas in *Dipsas* and *Sibynomorphus* it is divided in two distinct parts (Figures 1A,B,2A,B; Table 1). The divided infralabial gland in these two genera is composed of a thin stripe of gland that runs along the lip and below the infralabial scales, from the anterior tip of the dentary to the corner of the mouth (Figure 1A,B), and a much larger gland that runs along the ventrolateral surface of the mandible (Figures 1A,B, 2A,B).

**Table 1:** **Presence of the mandibular duct and type of insertion of both *levator anguli oris *** **(LAO) and *intermandibularis posterior pars posterior *** **(IPP) muscles in dissected species of the snail-eating dipsadine clade**

**Species**	**Mandibular duct**	**LAO insertion**	**IPP insertion**
*Dipsas albifrons*	Present	Anterior region of dentary	Connected to the gland
*Dipsas alternans*	Present	Anterior region of dentary	Connected to the gland
*Dipsas brevifascies*	Absent	Anterior region of dentary	Parallel to the gland
*Dipsas bucephala*	Present	Anterior region of dentary	Connected to the gland
*Dipsas catesbyi*	Present	Anterior region of dentary	Parallel to the gland
*Dipsas gracilis*	Present	Anterior region of dentary	Parallel to the gland
*Dipsas incerta*	Present	Anterior region of dentary	Parallel to the gland
*Dipsas indica*	Present	Anterior region of dentary	Connected to the gland
*Dipsas neivai*	Present	Anterior region of dentary	Connected to the gland
*Dipsas oreas*	Present	Anterior region of dentary	?
*Dipsas pakaraima*	Absent	Anterior region of dentary	Parallel to the gland
*Dipsas pavonina*	Present	Anterior region of dentary	Connected to the gland
*Dipsas peruana*	Present	Anterior region of dentary	Connected to the gland
*Dipsas sanctijoannis*	Present	Anterior region of dentary	Parallel to the gland
*Dipsas temporalis*	Absent	Anterior region of dentary	Parallel to the gland
*Dipsas tenuissima*	Absent	Anterior region of dentary	?
*Dipsas variegata*	Present	Anterior region of dentary	Connected to the gland
*Sibon annulatus*	Absent	Infralabial gland	Parallel to the gland
*Sibon carri*	Absent	Infralabial gland	Parallel to the gland
*Sibon dimidiatus*	Absent	Infralabial gland	Parallel to the gland
*Sibon nebulatus*	Absent	Infralabial gland	Parallel to the gland
*Sibon sanniolus*	Absent	Infralabial gland	Parallel to the gland
*Sibynomorphus garmani*	Present	Anterior region of dentary	?
*Sibynomorphus lavillai*	Present	Anterior region of dentary	Connected to the gland
*Sibynomorphus mikanii*	Present	Anterior region of dentary	Parallel to the gland
*Sibynomorphus neuwiedi*	Present	Anterior region of dentary	Parallel to the gland
*Sibynomorphus petersi*	Present	Anterior region of dentary	Connected to the gland
*Sibynomorphus turgidus*	Present	Anterior region of dentary	Parallel to the gland
*Sibynomorphus vagus*	Absent	Anterior region of dentary	Connected to the gland
*Sibynomorphus ventrimaculatus*	Present	Anterior region of dentary	Parallel to the gland
*Sibynomorphus williamsi*	Present	Anterior region of dentary	?
*Tropidodipsas fischeri*	Absent	Infralabial gland	Parallel to the gland
*Tropidodipsas sartorii*	Absent	Infralabial gland	Parallel to the gland

**Figure 3:**
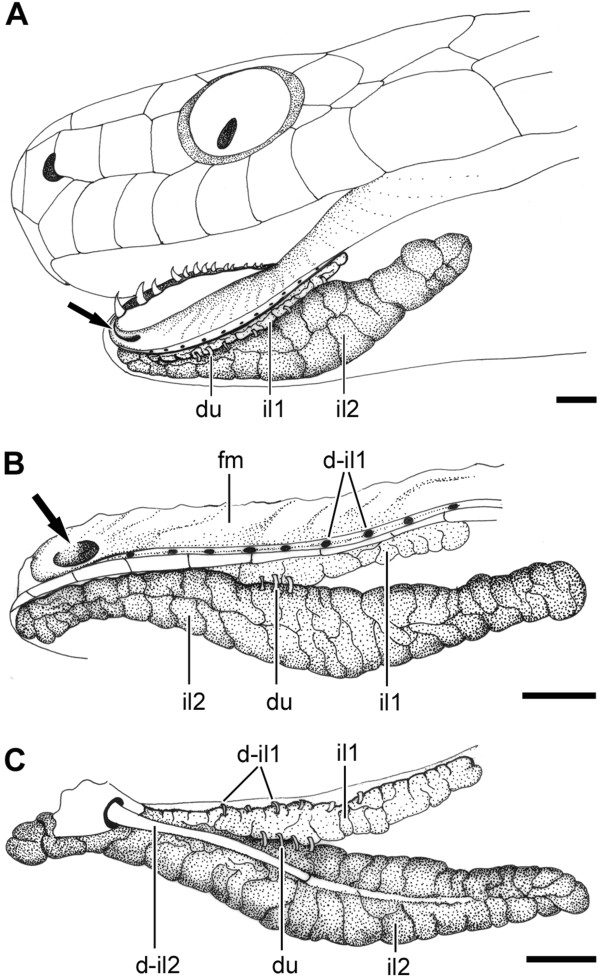
**Infralabial glands in Dipsadini.** Lateral view of the head of *Sibynomorphus turgidus* (IBSP 46431) **(A)**. Labial **(B)** and lingual **(C)** sides of il1 and il2 and associated buccal tissues after removal from the specimen. Abbreviations: du, ducts; d-il1, ducts of lateral, mucous infralabial gland; d-il2, duct of ventrolateral, seromucous infralabial gland; il1, lateral, mucous infralabial gland; il2, ventrolateral, seromucous infralabial gland; fm, floor of the mouth. Arrow points to the opening of the duct of il2 (d-il2, mandibular duct). Scale bar in all pictures = 1.25 mm.

We distinguish in the text below the more dorsolateral part of the infralabial gland from the more hypertrophied ventrolateral part, as the “mucous infralabial gland” (il1) and the “seromucous infralabial gland” (il2), respectively (Figure 3). In *Dipsas* and *Sibynomorphus*, the two portions of the infralabial gland are anteriorly connected through the glandular body and through a series of small ducts (Figure 3).

**Figure 4:**
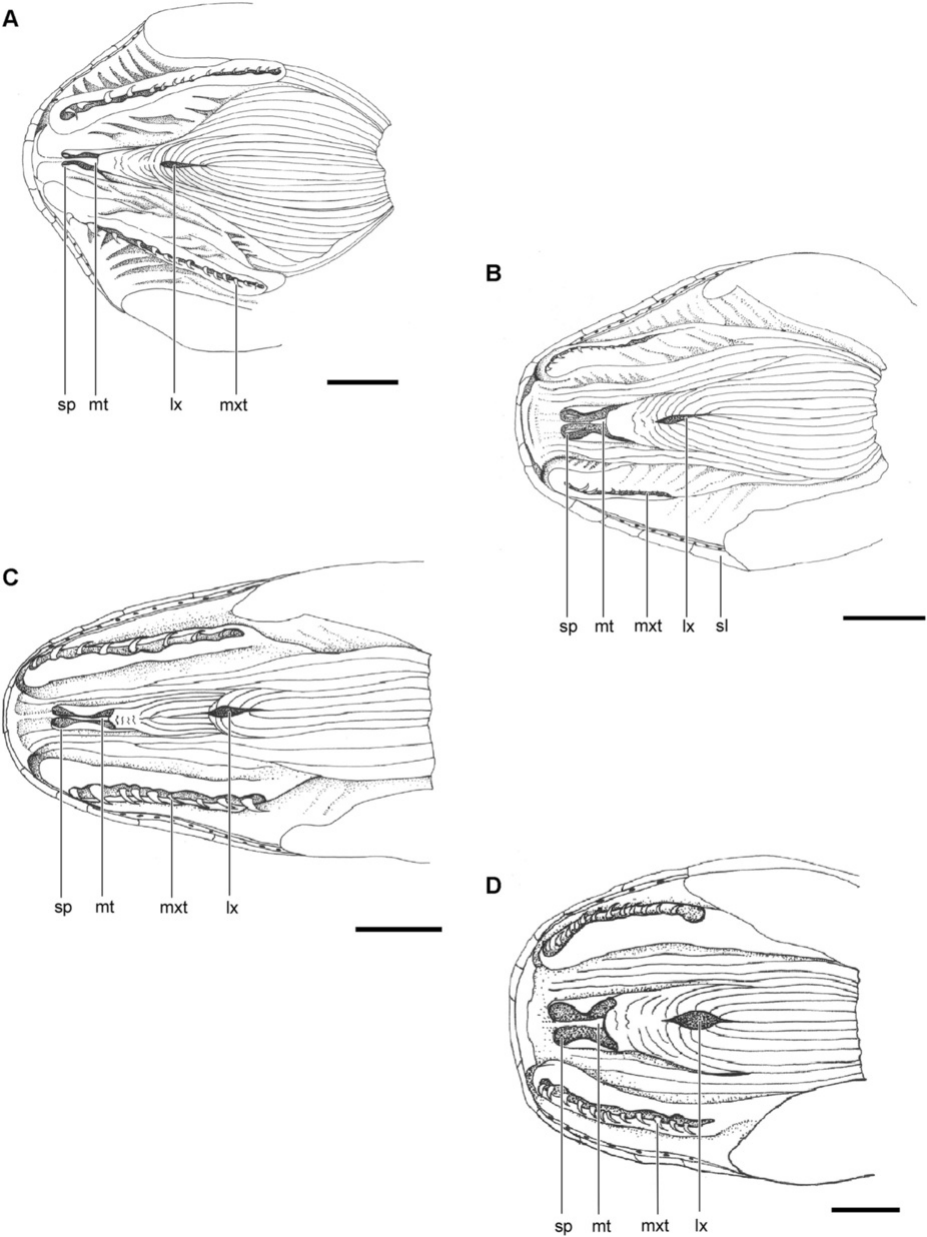
**Floor of the mouth in Dipsadini.***Dipsas neivai* (IBSP 70310) **(A)**, *Sibynomorphus mikanii* (IBSP 70224) **(B)**, *Sibon nebulatus* (MZUSP 6221) **(C)**, and *Tropidodipsas sartorii* (KU 157638) **(D)**. Abbreviations: lx, larynx; mt, median tubercle; mxt, maxillary tooth; sl, supralabial scale; sp, sublingual plica. Scale bar in pictures **A**-**B** = 5 mm and **C**-**D** = 2.5 mm.

**Figure 5:**
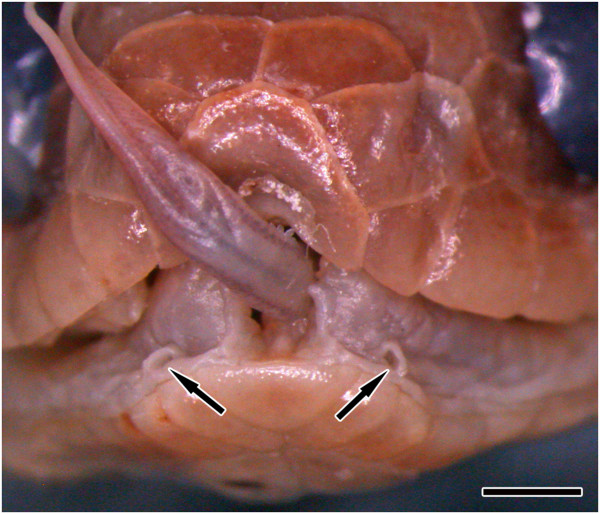
**Frontal view of the head of *Dipsas alternans*.** Openings of the mandibular ducts (d-il2), expanded and visible laterally to the tip of the dentaries (arrows) in *Dipsas alternans* (MZUSP 8833). Scale bar = 2 mm.

**Figure 6:**
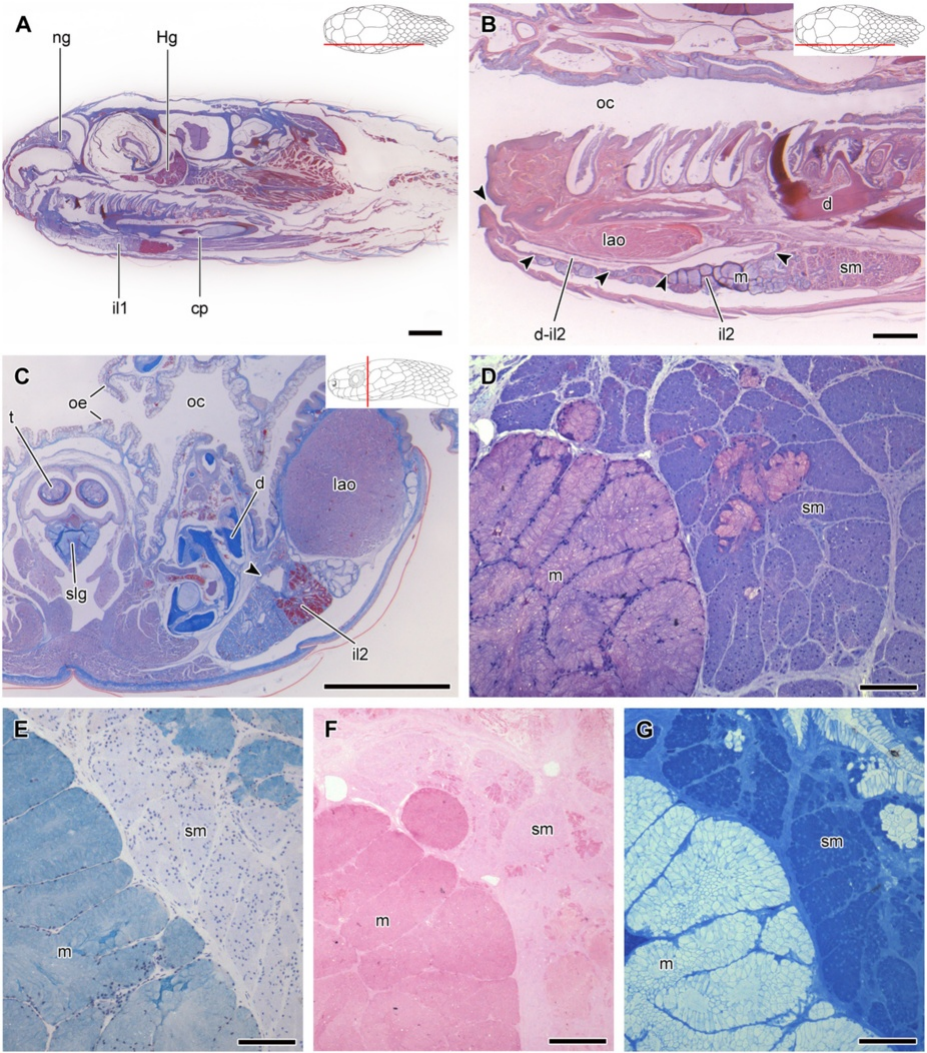
**Histological sections of the head of *Dipsas albifrons*.** Sagittal section of the head of *Dipsas albifrons* (MZUSP 17885); Paraffin section, Mallory trichrome staining **(A)**. Higher magnification focusing on the mandibular region and showing the difference in staining between the anterior and the posterior regions of il2, composed by mucous cells (m) and seromucous cells (sm), respectively. A large duct (d-il2) connects the posterior region of the seromucous infralabial gland (il2) to the floor of the mouth (arrowheads); Paraffin section, Hematoxylin-eosin staining **(B)**. Transverse section of the mandibular region showing contact between mucous and seromucous cells of the infralabial gland (il2) with its large duct (arrowheads) localized under the muscle *levator anguli oris*; Paraffin section, Mallory trichrome **(C)**. Limit between the anterior mucous cells (m) and posterior seromucous cells (sm) of the il2; Historesin, Toluidine blue-fuchsin **(D)**. Alcian blue, pH 2.5, histochemistry also revealing the highly positive result for the mucous cells (m). Nuclear staining with hematoxylin; Historesin **(E)**. PAS histochemistry revealing the highly positive result for the mucous cells (m); Historesin **(F)**. Bromophenol blue histochemistry revealing a positive reaction in the seromucous cells (sm), and contrasting with the negative result of the mucous cells (m); Historesin **(G)**. Abbreviations: cp, compound bone; d, dentary bone; d-il2, duct of ventrolateral, seromucous infralabial gland; Hg, harderian gland; lao, muscle *levator anguli oris*; ng, nasal gland; oc, oral cavity; oe, oral epithelium; slg, sublingual gland; t, tongue. Scale bar in pictures **A**-**C** = 1 mm and D-G = 100 μm. Panels at the upper right corner denote position of the section in **A**, **B**, **C**.

**Figure 7:**
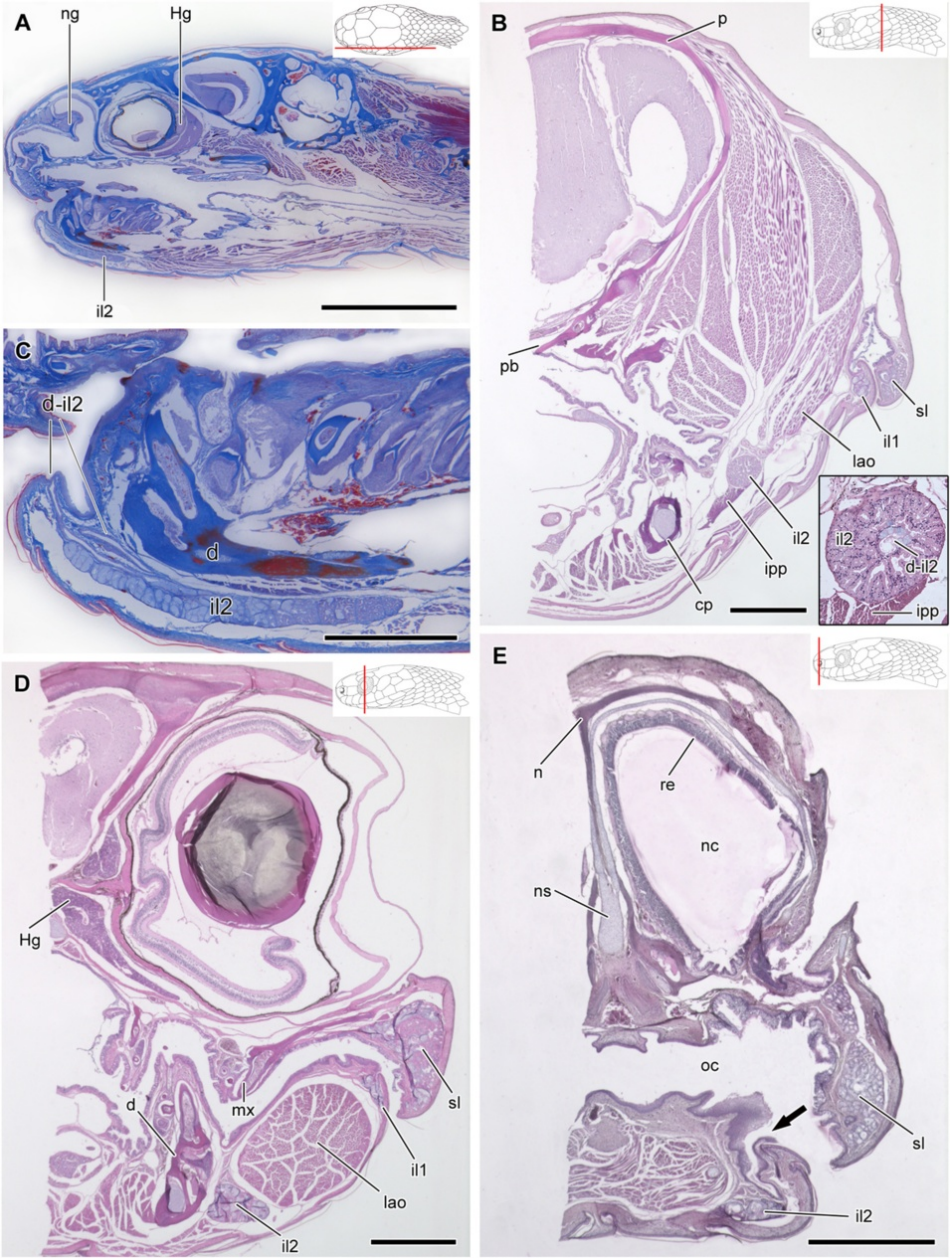
**Histological sections of the head of *Dipsas indica*.** Sagittal section of the head of *Dipsas indica* (IBSP 73451); Paraffin section, Mallory trichrome staining **(A)**. Transversal section of the posterior region of the head (MZUSP 16695) showing infralabial gland divided in two portions (il1 and il2) and muscle *intermandibularis posterior pars posterior* (ipp) associated with posteriormedial region of the il2 (shown in a higher magnification in the lower right corner); Paraffin, Hematoxylin-eosin staining **(B)**. Higher magnification of **(A)** focusing the anterior part of the mandibular region to show the duct and distinct portions of il2 constituted by mucous cells anteriorly and seromucous cells posteriorly. The duct of the il2 (d-il2) is connected to the anterior region of the mouth; Paraffin section, Mallory trichrome staining **(C)**. Transversal section of the head at the level of the eye (MZUSP 16695), showing the two portions of the infralabial gland (il1 and il2); Paraffin, Hematoxylin-eosin staining **(D)**. Transversal section of the head at the level of the snout (MZUSP 16695), showing opening of the duct (arrow) in the floor of the mouth; Paraffin, Hematoxylin-eosin staining **(E)**. Abbreviations: d, dentary; Hg, harderian gland; il1, lateral, mucous infralabial gland; il2, ventrolateral, seromucous infralabial gland; ipp, muscle *intermandibularis posterior pars posterior*; mx, maxillary; n, nasal; ng, nasal gland; ns, nasal septum; sl, supralabial gland; oc, oral cavity; p, parietal; pb, parabasisphenoid; re, respiratory epithelium. Scale bar in picture **A** = 5 mm; **B**, **D** and **E** = 500 μm; **C** = 1 mm. Panels at the upper right corner denote position of the section in **A**, **B**, **D**, **C**.

The epithelium of the floor of the mouth in Dipsadini is modified into a loose and extensively folded epithelial tissue that covers the whole oral cavity, being more accentuated in *Dipsas* and *Sibynomorphus* where a heavily folded epithelium accommodates the large muscle *levator anguli oris* laterally to the dentary (Figure 4). The floor of the mouth in most species of *Dipsas* and *Sibynomorphus* examined is also apparently unique in having a pair of openings at the level of the intermandibular raphe for the release of secretion coming from il2 through a single large duct (Figure 5; Table 1), also revealed by histological sections in *D. albifrons* and *D. indica* (Figures 6 and 7). We failed to confirm the presence of “mandibular ducts” in six species of *Dipsas* (*D. brevifascies*, *D. pakaraima*, *D. temporalis*, *D. tenuissima*) and one *Sibynomorphus* (*S. vagus*), although their presence could not be completely ruled out (Table 1).

The il1 of *Dipsas* and *Sibynomorphus* and the single infralabial gland of *Sibon* and *Tropidodipsas* are similarly connected with the mouth by short ducts along the margin of the lower lip, as in all other Dipsadinae. The short openings of the ducts of the il1 are similar in size and proportion in all Dipsadinae, except in *Sibynomorphus* and *Crisantophis* where they are significantly larger in diameter. The ducts of the il1 in Dipsadinae open inside a shallow gutter, or fold, that runs along the lower lip and sets the boundary between the soft epithelium of the mouth and the cornified infralabial scales (Figure 3). This fold is much more pronounced in all four genera of Dipsadini, and forms a deep gutter that tends to close dorsally by the contact of both margins. In *Sibon* and *Sibynomorphus*, a second deep fold with no ducts runs parallel to the latter, forming a double infolding along the margin of the lip (Figure 3) that was not observed in *Dipsas* and *Tropidodipsas*.

In all four genera of Dipsadini, the median tubercle is reduced in size, forming only a small, anteriorly tapering protuberance. The sublingual plicae are also poorly developed on the floor of the mouth and closely approach each other anteriorly due to the reduction of the median tubercle. The outer tongue sheath is reduced, and delineates a narrow opening for the tongue in *Dipsas*, while it forms a larger opening for the tongue in *Sibon*, *Sibynomorphus*, and *Tropidodipsas* (Figure 4). *Sibon nebulatus* differs from the other species analyzed in respect to the position of the larynx that is located more posteriorly on the floor of the mouth, being separated from the outer sheathing tongue by at least twice the distance than in other dipsadines (Figure 4C).

### Histology and histochemistry of the infralabial glands of Dipsadini

Histological procedures were performed in adult individuals representing four genera of snail-eating snakes (*Dipsas*, *Sibon*, *Sibynomorphus*, *Tropidodipsas*) (see Additional file 2).

**Figure 8:**
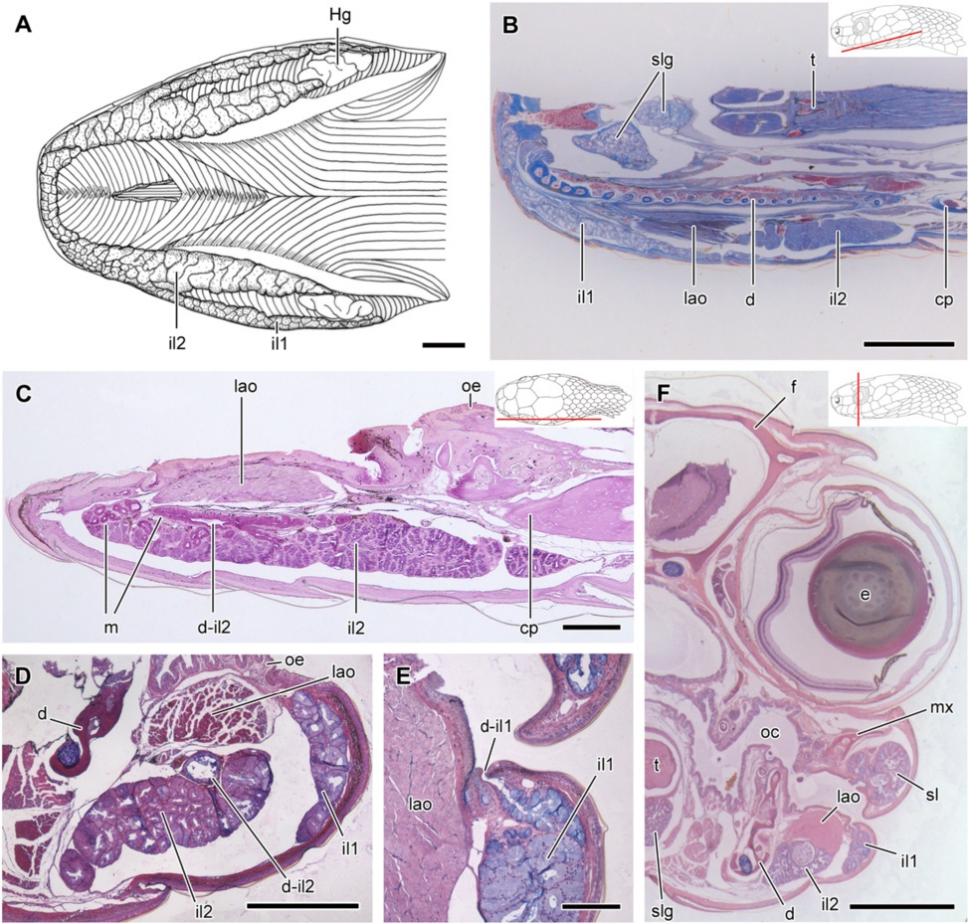
**Histological sections of the head of *Sibynomorphus mikani and S. neuwiedi*.** Ventral view of the skinned head of *Sibynomorphus mikanii* evidencing the size and location of both il1 and il2; Paraffin section, Mallory trichrome staining **(A)**. Sagittal section of the head of *Sibynomorphus mikanii* showing the position of the two portions of the infralabial gland (il1 and il2); Paraffin section, Mallory trichrome staining **(B)**. PAS histochemical reaction in a longitudinal section of the mandibular region of *Sibynomorphus mikanii* (MZUSP 17886) revealing the more developed portion of infralabial gland (il2) with the duct (d-il2) running towards the anterior region. Although the whole gland reacts to PAS, mucous cells (m) in the anterior region and the duct (d-il2) are much more positive; Paraffin section **(C)**. Transverse section of the mandibular region of *Sibynomorphus mikanii* (MZUSP 17882) showing the infralabial gland divided in il1 and il2, and evidencing the duct of il2 (d-il2); Paraffin section, Hematoxylin-eosin staining **(D)**. Transverse section of the head of *Sibynomorphus neuwiedi* (MZUSP 17225) evidencing a duct in il1; Paraffin section, Hematoxylin-eosin staining **(E)**. Transverse section of the head of *Sibynomorphus neuwiedi* showing il1 and il2 separated by the bundle of the muscle *levator anguli oris*; Paraffin section, Hematoxylin-eosin staining **(F)**. Abbreviations: cp, compound bone; d, dentary bone; d-il1, ducts of the lateral, mucous infralabial gland; d-il2, duct of the ventrolateral, seromucous infralabial gland; e, eye; f, frontal; Hg, Harderian gland; il1, lateral, mucous infralabial gland; il2, ventrolateral, seromucous infralabial gland; lao, muscle *levator anguli oris*; m, mucous cells; mx, maxillary; oc, oral cavity; oe, oral epithelium; sl, supralabial gland; slg, sublingual gland; t, tongue. Scale bar in pictures **A**-**D** and **F** = 1 mm and **E** = 50 μm. Panels at the upper right corner denote position of the section in **B**, **C**, **F**.

**Figure 9:**
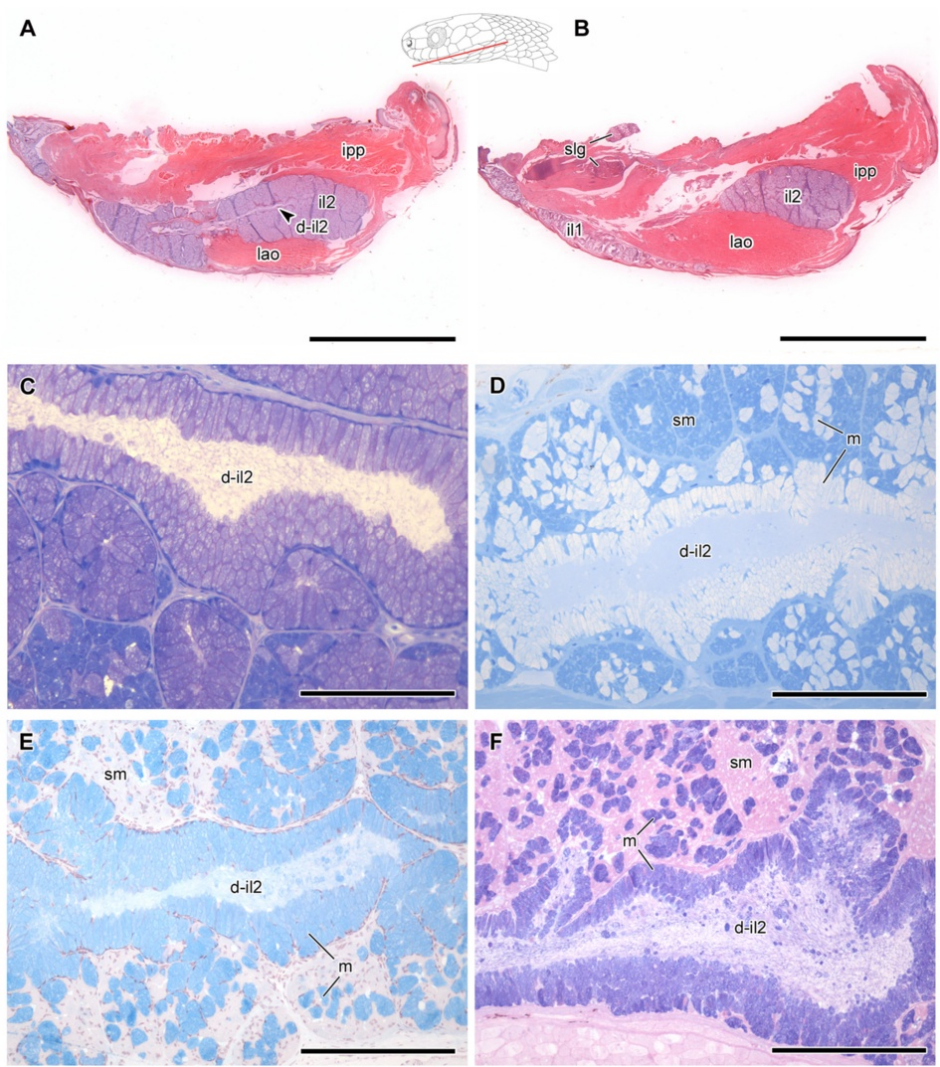
**Histological sections of the infralabial glands of *Dipsas neivai*.** Two distinct horizontal planes from serial histological sections of the head of *Dipsas neivai* (MZUSP 14665) showing part of the larger infralabial gland (il2) with the duct (d-il2) in the central area **(A)**, and part of the smaller and thinner mucous infralabial gland (il1), extending along the margin of the lip **(B)**, and their relationship with muscles *levator anguli oris* (lao) and *intermandibularis posterior pars posterior* (ipp). While the larger seromucous infralabial gland (il2) is embraced by both muscles, the thinner gland (il1) is connected only with the muscle *levator anguli oris*; Paraffin sections, Hematoxylin-eosin staining **(A-B)**. Longitudinal historesin sections of il2 focusing the duct (d-il2) and the surrounding acini **(C-F)**. Toluidine blue-fuchsin **(C)**. Bromophenol blue histochemical reaction, indicating a positive result in most parts of the cells that form the acini, characterizing their seromucous condition (sm) **(D)**. Alcian blue pH 2.5 histochemical reaction revealing acid mucous cells (m) within the acini and in the duct of ventrolateral, seromucous infralabial gland (d-il2); Nuclear staining with hematoxylin **(E)**. Alcian blue pH 2.5 + PAS, confirming the result shown in **D** and **E(F)**. Abbreviations: il1, lateral, mucous infralabial gland; il2, ventrolateral, seromucous infralabial gland; slg, sublingual gland; m, mucous cells; sm, seromucous cells. Scale bar in pictures **A**-**B** = 1.5 mm and **C**-**F** = 100 μm. Panel at the upper right corner of **A** denotes position of the sections in **A** and **B**.

Infralabial glands are basically composed by the secretory portion forming the glandular body and by ducts in its interior. They are enveloped by a thin layer of connective tissue that penetrates the glandular body, dividing the gland in lobuli and involving all acini and ducts (Figures 8C and 9C). Due to the spatial arrangement of the two portions of the infralabial gland (il1 and il2) in the mandible, they are rarely seen simultaneously in a single histological section, justifying the need for serial sections in sagittal and horizontal planes that allow a three-dimensional interpretation of the structures.

In *Dipsas albifrons*, the posterior region of the il2 is composed only by seromucous cells, while the rest of the gland is composed of mucous cells (Figure 6A,B). The limit between these two regions is clearly visible through the contrasting stain of seromucous and mucous cells (Figure 6D). While seromucous cells react weakly positive to PAS and highly positive to bromophenol blue (Figure 6F and G, respectively), mucous cells show an intense positive reaction to alcian blue pH 2.5 and PAS (Figure 6E,F). The same pattern of cellular distribution is observed in *D. indica*[10] (Figure 7A,C). Sections of the heads of both *D. albifrons* and *D. indica* reveal the presence of the mandibular duct that runs along the longitudinal middle of the il2, extending posteroanteriorly to open in the floor of the mouth at the level of the intermandibular raphe (Figures 6B,C and 7B,C and E). The duct is lined with a simple epithelium constituted by columnar mucous cells.

In *Sibynomorphus mikanii*, the il2 shows a simple epithelium with polygonal cells forming acini (Figure 8C). The lumen of these acini is very narrow and difficult to observe, being filled with secretion in the few histological sections where the structure is visible. The mandibular duct is clearly visible in a sagittal section of the lower jaw of *S. mikanii* (Figure 8C). The il2 of *S. mikanii* reacts positively to bromophenol blue. In *S. mikanii* and *S. neuwiedi*, the il1 shows a simple epithelium, with acini constituted mainly by mucous cells (Figure 8B,D,E). As shown in transversal sections of the head of *S. neuwiedi*, the il1 presents a series of short ducts that open just under the infralabial scales (Figure 8E,F).

*Dipsas neivai* also presents a large mandibular duct that runs along the longitudinal middle of the il2 (Figure 9A). However, none of the available sections provided a clear view of the opening of the duct at the level of the mouth floor. The il2 is composed by prismatic secretory cells arranged in acini, and its mandibular duct is lined by a simple columnar epithelium (Figure 9C). Cells lining both il1 and il2 ducts are always of mucous nature, reacting positively to PAS and alcian blue pH 2.5 (Figure 9E,F), while part of the cells forming acini react positively to bromophenol blue (Figure 9D), revealing their seromucous nature and another part is mucous, reacting positively to alcian blue pH 2.5 (Figure 9E).

**Figure 10:**
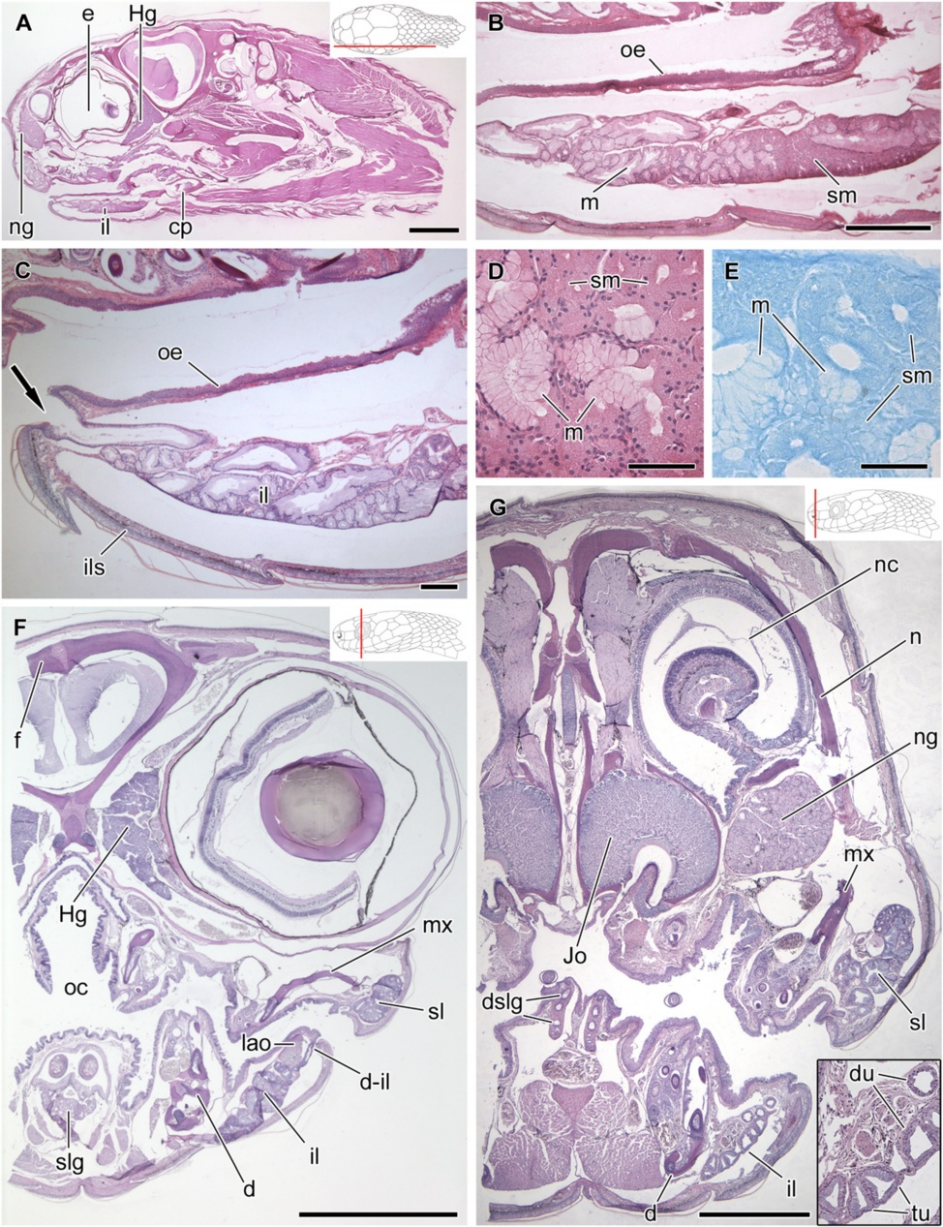
**Histological sections of the head of *Sibon nebulatus.*** Sagittal section of the head of *Sibon nebulatus* (MZUSP 9316); Paraffin, Hematoxylin-eosin staining **(A)**. Mandibular region showing infralabial gland constituted by mucous (m) and seromucous cells (sm); Paraffin, Hematoxylin-eosin staining **(B)**. Detail of the duct from infralabial gland opening in the oral cavity; Paraffin, Hematoxylin-eosin **(C)**. Epithelium of the infralabial gland constitute by mucous (m) and seromucos cells (sm); Paraffin, Hematoxylin-eosin staining **(D)**. Histochemical reaction of the bromofenol blue showing positivity for the posteriormost cells of the infralabial glands; Paraffin **(E)**. Transversal section of the head at the level of the eyes showing infralabial gland (il) and its association with the muscle *levator anguli oris* (lao); Paraffin, Hematoxylin-eosin staining **(F)**. Transversal section of the head at the level of the snout showing infralabial glands with its tubules (tu) and ducts (du) distended to the anterior portion of the mouth; Paraffin, Hematoxylin-eosin staining **(G)**. Abbreviations: cp, compound bone; d, dentary; d-il, ducts of the infralabial gland; dslg, ducts of the sublingual gland; e, eye; f, frontal; Hg, Harderian gland; ils, infralabial scales; Jo, Jocobson’s organ; lao, muscle *levator anguli oris*; mx, maxillary; n, nasal bone; nc, nasal cavity; ng, nasal gland; oc, oral cavity; oe, oral epithelium; sl, supralabial gland; slg, sublingual gland. Scale bar = 1 mm **(A, F and G)**; 500 μm **(B)**; 200 μm **(C and E)**; 50 μm **(D)**. Panels at the upper right corner denote position of the section in **A**, **F**, **G**.

In *Sibon nebulatus*, the infralabial gland is constituted by mucous and seromucous cells organized in tubules and acini (Figure 10A,B). The acini are observed in the posterior most portion of the gland and are mainly constituted by seromucous cells, being more intensively stained by hematoxylin-eosin and bromofenol blue. On the other hand, the tubules are mainly constituted by mucous cells that stain only with hematoxylin-eosin (Figure 10B,D,E). In the central region of the gland, a series of ducts extend along the glandular body, opening in the anterior region of the mouth (Figure 10C,G). In addition to these large ducts, a series of shorter ducts are arranged perpendicularly to the gland, opening more posteriorly, between the infralabial scales and the oral epithelium. Posteriorly, at the level of the insertion of the LAO into the gland, the ducts surround the muscle to reach the oral epithelium (Figure 10F).

**Figure 11:**
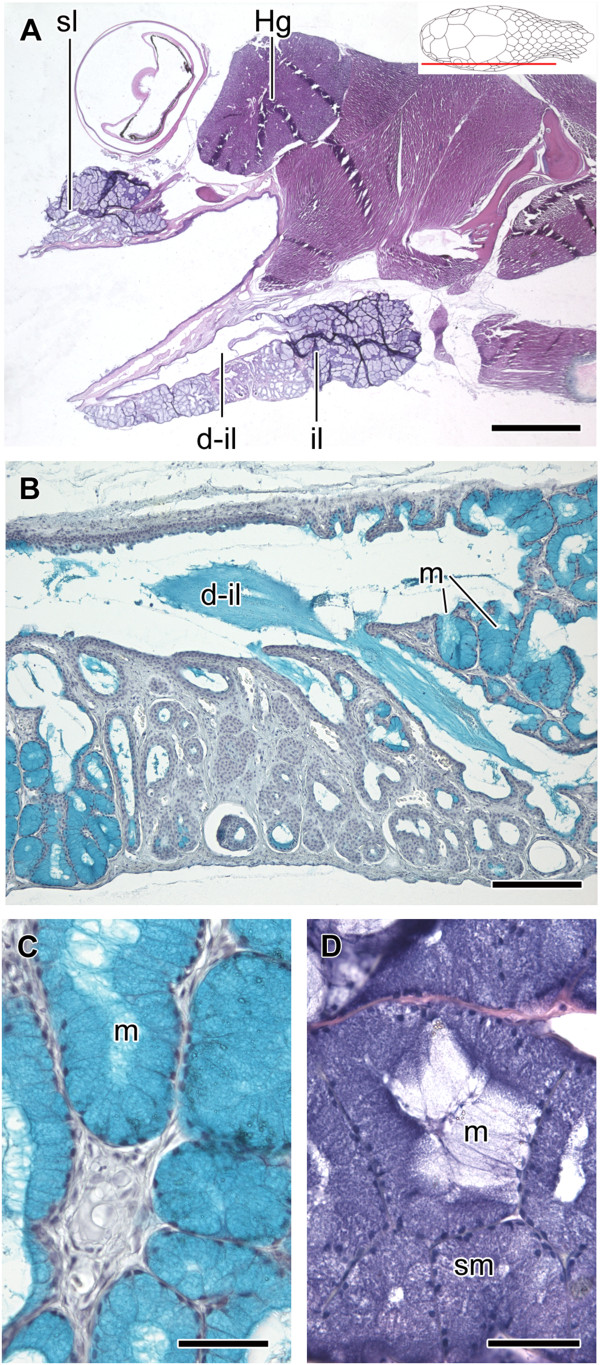
**Histological sections of the head of *Tropidodipsas sartorii*.** Sagittal section of the head of *Tropidodipsas sartorii* (USNM 561067) showing supra (sl) and infralabial (il) glands, and emphasizing the large duct (d-il) distended along the medial surface of the infralabial gland; Paraffin, Hematoxylin-eosin staining **(A)**. Alcian blue pH 2.5 reaction showing mucous cells (m) and the large duct (d-il) distended in medial surface of the infralabial gland; Paraffin **(B)**. Detail of the acini of the infralabial gland and its positivity to alcian blue pH 2.5; Paraffin **(C)**. Acini in the posteriormost portion of the gland constituted by seromucous cells (sm) strongly stained by hematoxylin-eosin and mucous cells (m) slightly stained **(D)**. Abbreviations: Hg, Harderian gland; sl, supralabial gland. Scale bar = 1 mm **(A)**; 200 μm **(B)**; 50 μm **(C and D)**. Panel at the upper right corner denote position of the section in **A**.

In *Tropidodipsas sartorii*, the infralabial gland is mainly constituted by mucous cells that are arranged in acini and distributed along the whole body of the gland (Figure 11A). These mucous cells stain with hematoxylin-eosin and react positively to alcian blue pH 2.5 (Figure 11A,C). Only the posteriormost region of the gland presents a series of small acini that are composed by seromucous cells that stain only with hematoxylin-eosin (Figure 11D). The infralabial gland of *T. sartorii* has a large duct that extends along its medial surface, reaching its anterior portion (Figure 11A). This duct is formed by the confluence of a series of shorter and smaller converging ducts and does not correspond to the mandibular duct of il2 (Figure 11A,B). Both larger and smaller ducts are constituted by cells with low cytoplasm and several nuclei, resembling a stratified epithelium (Figure 11B).

### Muscles associated with the infralabial glands of Dipsadini

In all Dipsadini, both *levator anguli oris* (LAO) and *intermandibularis posterior pars posterior* (IPP) muscles were observed to be closely associated with the infralabial glands, being adpressed to the wall of the gland or attached to it as a compressor of the gland (Table 1). The relation between these muscles and the infralabial glands il1 and il2, the lower jaw, and the corner of the mouth are described below.

The LAO of Dipsadini is always functionally and morphologically distinct from the *adductor mandibulae externus superficialis* muscle (AES; *sensu* Zaher [28]), and are treated here as two separate muscular units [28]. The well-developed LAO of goo-eating snakes is innervated by its own anterior branch of the ramus mandibularis of the trigeminal nerve [28]. Among all *adductores externi* muscles, only the LAO is directly associated with the infralabial glands in snail-eating dipsadines.

The LAO of Dipsadini is a long, parallel fibered muscle that extend from behind the eye to the anterior half of the lower jaw, curving around the angle of the mouth to insert on the mandible or on the surface of the il1. The anterolateral surface of the epimysium is always firmly attached to the buccal membrane of the corner of the mouth. Except for these few characteristics, the LAO showed significant variation among studied genera, being completely differentiated from the AES in all goo-eaters, except in *Tropidodipsas*, *Sibon*, and *Atractus* in which some of the more posterodorsal fibers tend to share an aponeurosis with the more anteromedial fibers of the AES (Figure 1).

**Figure 12:**
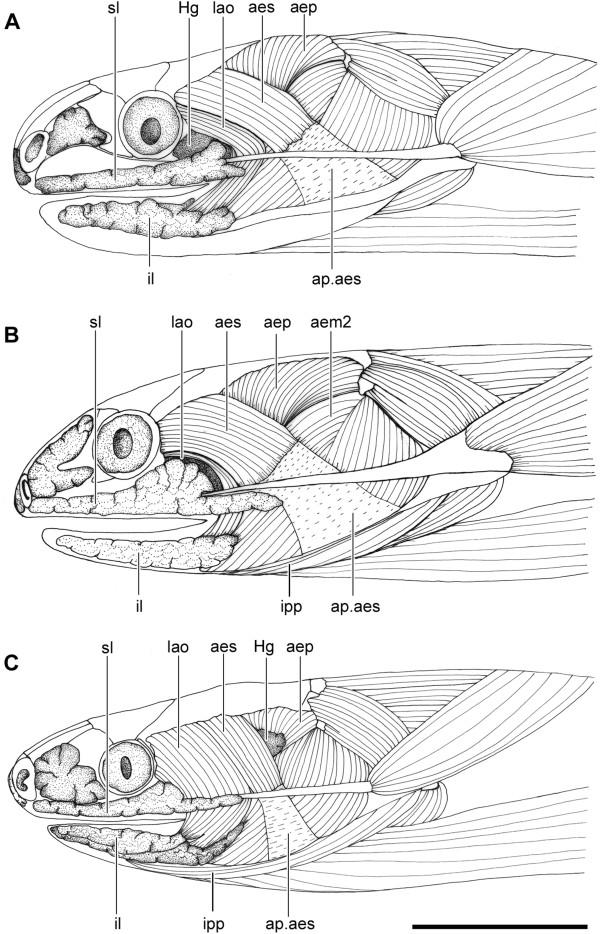
**Head muscles and glands of Dipsadinae.** Lateral view of the head of *Atractus major* (IBSP 43395) **(A)**, *Adelphicos veraepacis* (KU 187320) **(B)**, and *Geophis zeledoni* (KU 63822) **(C)**, showing location of the infralabial gland (il) with respect to the head muscles and mandible. Abbreviations: aem2, muscle *adductor mandibulae externus medialis*; aep, muscle *adductor mandibulae externus profundus*; aes, muscle *adductor mandibulae externus superficialis*; ap.aes, aponeurose of muscle *adductor mandibulae externus superficialis*; Hg, harderian gland; ipp, muscle *intermandibularis posterior pars posterior*; lao, muscle *levator anguli oris*; sl, supralabial gland. Scale bar in all pictures = 5 mm.

The LAO is anterior to the AES in *Dipsas*, *Sibynomorphus*, and *Atractus*. In two species of *Atractus* (*A. major* and *A. flammigerus*), the posteriormost fibers of the LAO are medial to the AES (Figure 12A). In *Ninia*, *Chersodromus*, and *Geophis*, the anterior half of the LAO is anterior to the AES while the posterior fibers broadly overlap the anterior half of the AES at their origin. The LAO is medial in *Sibon* and *Adelphicos* (Figures 1C, 12B). In *Tropidodipsas*, the two species examined showed distinct arrangements, the LAO being lateral to the AES in *T. fischeri* and medial in *T. sartorii* (Figure 1)*.*

In all species of *Dipsas* examined, the origin of the LAO is very large, encompassing the entire postocular region laterally. Three distinct conditions are found in *Dipsas*. In *Dipsas incerta*, *D. brevifascies*, and *D. temporalis*, the LAO arises from the entire laterodorsal surface of the postorbital. The postorbital bone is long, reaching the ectopterygoid ventrally. In *Dipsas bucephala*, *D. indica*, *D. catesbyi*, and *D. albifrons*, the origin of the LAO extends from the base of the postorbital to the lateral tip of the maxillary ramus of the ectopterygoid. *Dipsas neivai* shows the largest condition, with the LAO extending from the base of the postorbital to the posterolateral edge of the maxilla, and also encompassing the lateral tip of the maxillary ramus of the ectopterygoid. The postorbital being vestigial in *D. neivai*, the origin of the LAO is mostly on the posterior surface of a well-developed maxillo-postorbital ligament (Figure 1A).

The LAO integrally covers the Harderian gland, except in *D. brevifascies* where the gland is visible ventrally to the LAO. After curving the angle of the mouth, the LAO extends anteriorly to reach the anteriormost region of the dentary where the bundle ends in a broad aponeurosis that attaches to the ventrolateral edge of the anterior one-third of the dentary. The muscle does not insert broadly on the fascia of the infralabial gland, although it is somewhat tied by its fascia to the dorsal surface of the gland. In all species of *Dipsas* examined, the ventral (mandibular) portion of the LAO is visible dorsally to the il2, except in *D. brevifascies* where the muscle is completely covered by the infralabial gland. At the level of the anterior one third of the dentary, the il2 expands to the labial edge, covering the anterior tip of the bundle and its aponeurosis.

The LAO in *Sibynomorphus* is also greatly developed. In two of the eight species examined (*S. mikanii* and *S. neuwiedi*), the LAO arises via a large aponeurosis from the anterior half of the dorsolateral ridge of the parietal, extending to the distal tip of the postorbital and lateral tip of the maxillary ramus of the ectopterygoid (Figures 1, 2). The Harderian gland is visible medially to the aponeurosis in both species. In *S. ventrimaculatus*, the aponeurotic sheet is reduced and the fibers of the LAO cover completely the Harderian gland. In *S. vagus* and *S. turgidus*, the origin of the LAO is less extensive, arising only from the base of the postorbital and extending ventrally to the lateral tip of the maxillary ramus of the ectopterygoid. As in *Dipsas*, the LAO of *Sibynomorphus* curves around the corner of the mouth, forming a funnel-shaped bundle that inserts via an extensive aponeurosis to the ventrolateral edge of the anterior one-third of the dentary.

The LAO differs greatly in the two species of *Tropidodipsas* examined. In *T. sartorii*, the origin of the muscle is medial to both AES and Harderian gland (Figure 1D). On the other hand, *T. fischeri* shows the opposite condition, with a LAO clearly lateral to the AES and Harderian gland. In the former species, the origin of the muscle is confined to a narrow area; the fibers arise from the posteromedial base of the postorbital and adjacent surface of the parietal, below the anterior fibers of the AES. Both posterolateral fibers of the LAO and anteromedial fibers of the AES arise from a short aponeurosis. After originating medially to the AES, the LAO of *T. sartorii* takes an anterior position to the latter, extending ventrally towards the corner of the mouth (Figure 1D). In *T. fischeri*, the LAO arises from the anteriormost surface of the dorsolateral crest of the parietal, extending on the dorsolateral surface of the proximal half of the postorbital. The posteriormost fibers of the LAO and the anterior fibers of the AES arise from a common aponeurosis. In both species, the LAO forms a fusiform bundle at the level of the corner of the mouth that curves around the latter and inserts on the dorsal surface of the posterior third of the infralabial gland. Some of the more dorsal fibers are longer and converge to insert on a thin tendon that extends anteriorly in parallel to the dorsal edge of the infralabial gland. The tendon attaches to the skin below the infralabial scales.

The position of the LAO in *Sibon* is virtually the same as in *Tropidodipsas sartorii*, except in *S. sanniolus* in which the more lateral and anterior fibers arise from an aponeurosis shared with the anterolateral fibers of the AES and from the lateral surface of the postorbital, respectively. These fibers are thus anterior to the AES. However, the medial fibers that represent most of the mass of the LAO in *S. sanniolus*, remain medial to the AES.

In all four genera of Dipsadini examined, the IPP originates on the lateral surface of the compound bone, at the level of the mandibular fossa. It is a thin muscle that extends anteriorly, superficial to the *neurocostomandibularis* muscle and in parallel to the mandible, to attach to the skin lateral to the mental region but medially to the infralabial gland (Figure 2). Both bundles of the IPP do not meet on the midline ventrally. Instead, they are closely associated to the fascia of the infralabial gland, in which the more lateral epimysium of the muscle attaches. In a few specimens of *D. neivai*, a significant part of the muscle overlaps the infralabial gland and may act as a compressor of the gland since the epimysium of the muscle tends to fuse with the fascia covering the gland. In both *Dipsas* and *Sibynomorphus* (except *S. neuwiedi*), in which the mental groove is lost, we observe an unusual condition of the bundles pertaining to the muscle *intermandibularis anterior pars posterior*, which are divided in small groups of fibers that are interlaced at the level of the ventral midline (Figure 2A,B).

### The muscles LAO and IPP in the remaining goo-eating snake genera *Adelphicos*, *Atractus*, *Geophis*, *Ninia*, and *Chersodromus*

In all species of *Atractus* examined, the LAO is anterior to the AES, except for the posteriormost fibers arising from the dorsolateral ridge of the parietal that are medial to the AES (Figure 12A). The LAO is a thin band of muscle arising from a narrow site of origin, which encompasses the posterolateral edge of the proximal half of the postorbital and anteriormost portion of the dorsolateral ridge of the parietal, just posterior to the postorbital-parietal contact. The LAO extends on a ventral and slightly posterior direction, curving around the corner of the mouth and inserting on the dorsal surface of the posterior third of the infralabial gland.

In *Adelphicos veraepacis*, the origin of the LAO is medial to the AES, arising from the anteriormost portion of the dorsolateral ridge of the parietal and the posterolateral margin of the short postorbital. The muscle extends ventrally and slightly posteriorly as a thin band of fibers, curving around the corner of the mouth and inserting on the dorsal surface of the posterior third of the infralabial gland (Figure 12B).

In all species of *Geophis* examined, the LAO corresponds to a thin, triangular sheet of muscle with an origin on the dorsolateral ridge of the parietal and postorbital, from the anterior edge of the muscle *adductor mandibulae externus profundus* (AEP; *sensu* Zaher, [28]) to the tip of the postorbital (Figure 12C). The posterior half of the LAO arises via an aponeurosis that overlies the dorsal portion of the AES. The fibers are directed dorsoventrally, converging to form a fusiform bundle at the level of the corner of the mouth. The site of insertion varies among the species examined. In *G. anocularis, G. rhodogaster*, *G. dubius*, and *G. hoffmanni*, the muscle terminates in an aponeurosis that inserts on the buccal membrane and lateral surface of the dentary dorsomedially to the infralabial gland. The epimysium of the LAO tends to be firmly attached to the dorsal surface of the posterior region of the infralabial gland. In *G. brachycephalus* and *G. zeledoni*, the muscle inserts broadly on the dorsal and dorsomedial surfaces of the posterior region of the infralabial gland (Figure 12C). The variation regarding the insertion site of the LAO seems to be correlated with the glandular differentiation present on the posterior region of the infralabial gland. In *G. brachycephalus* and *G. zeledoni*, where the LAO inserts on the infralabial gland, the gland shows two distinct anterior and posterior regions externally that are easily distinguished by their color and celular types.

**Figure 13:**
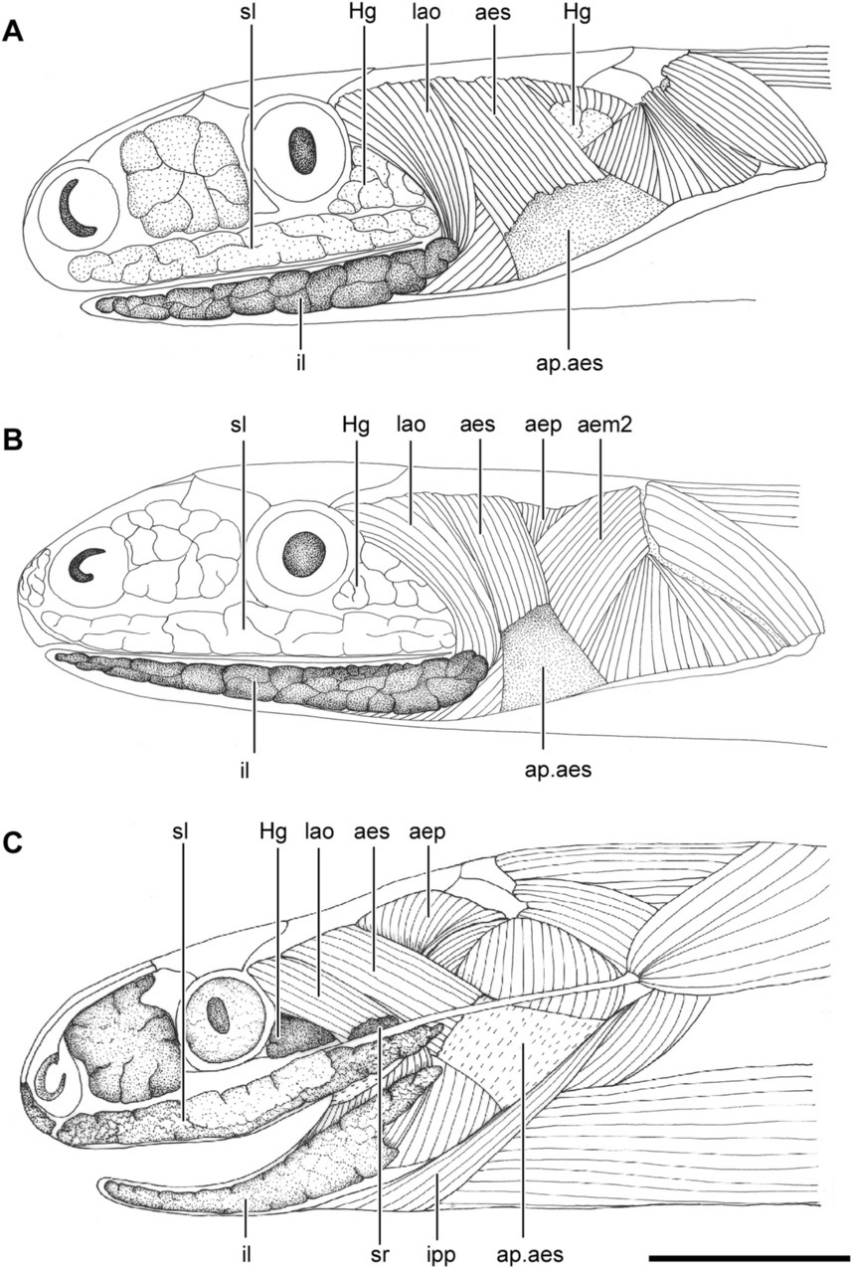
**Head muscles and glands of Dipsadinae.** Lateral view of the head of *Chersodromus liebmanni* (USNM 109932) **(A)**, *Enulius flavitorques* (KU 174188) **(B)**, and *Ninia atrata* (AMNH 59426) **(C)**, showing the location of infralabial gland (il) with respect to the head muscles and mandible. Abbreviations: aes, muscle *adductor mandibulae externus superficialis*; aem2, muscle *adductor mandibulae externus medialis*; aep, muscle *adductor mandibulae externus profundus*; ap.aes, aponeurose of muscle *adductor mandibulae externus superficialis*; Hg, harderian gland; ipp, muscle *intermandibularis posterior pars posterior*; lao, muscle *levator anguli oris*; n, nasal gland; pm, premaxillary gland; sl, supralabial gland; sr, superior rictal gland. Scale bar in all pictures = 5 mm.

The LAO of *Ninia* resembles the condition found in *Geophis*, with the significant difference that the muscle does not insert on the infralabial gland (except in one specimen of *N. maculata* and one of *N. atrata* in which the more lateral fibers insert on the dorsolateral surface of the gland). The LAO is a thin band of muscle originating on the dorsolateral ridge of the parietal and postorbital, from the middle of the AES to the proximal dorsolateral surface of the postorbital. The posterior half of the muscle overlaps the anterior half of the dorsal portion of the AES. Fibers extend ventrally to curve around the corner of the mouth and converge to terminate on a broad aponeurosis that attaches to the lateral surface of the dentary. The LAO of *Chersodromus* shows the same condition of *Ninia* (Figure 13A,C).

In the goo-eating snakes *Adelphicos*, *Atractus*, *Geophis*, *Ninia*, and *Chersodromus*, the IPP originates on the lateral surface of the compound bone, passing anteromedially as a thin bundle to attach to the ventral surface of the skin posteriorly to the mental region, with only some fibers reaching their counterpart at the midline. The epimysium of the muscle does not contact the infralabial gland.

### Phylogenetic relationships of Dipsadinae

Our hypothesis of dipsadine interrelationships is based on Maximum Likelihood (ML) and Bayesian (BI) analyses of 576 sequences from five mitochondrial and six nuclear genes in 87 terminal taxa (including 26 outgroup and 61 ingroup taxa; see Materials and Methods). Our study included a substantially denser sample of dipsadine species compared to previous ones [29-31]. The extended dipsadine sampling is meant to provide a thorough test of the monophyly of the tribe Dipsadini, thereby offering a more rigorous background for the resulting hypothesis of interrelationships of its constituent parts.

**Figure 14:**
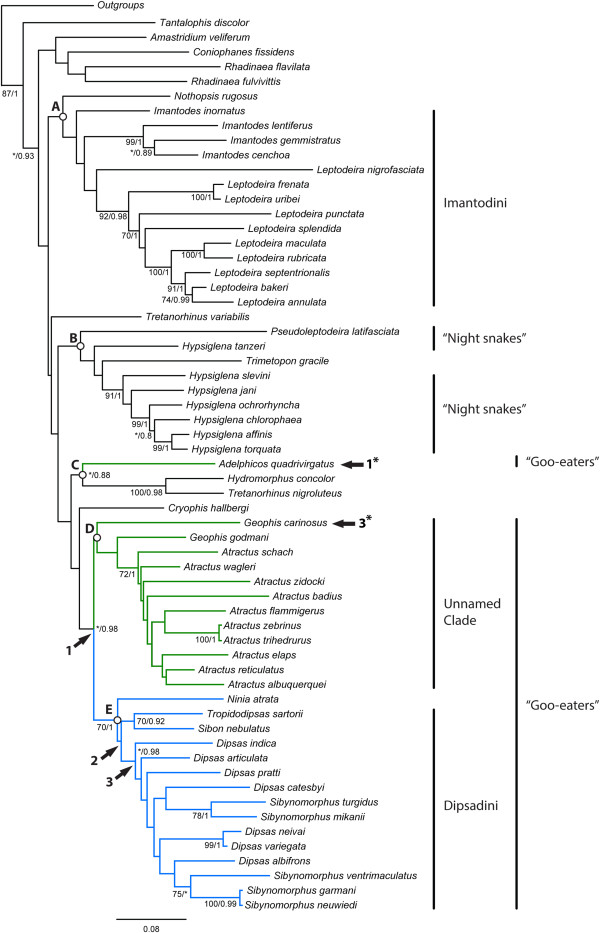
**Maximum likelihood tree of Dipsadinae.** Numbers at nodes show bootstrap and posterior probability support values retrieved in the maximum likelihood and Bayesian analyses, respectively. Asterisks indicate bootstrap support values lower than 70%. Clades and numbers on nodes are explained in the text. Numbers with an asterisk indicate homoplasy (see text for details).

**Figure 15:**
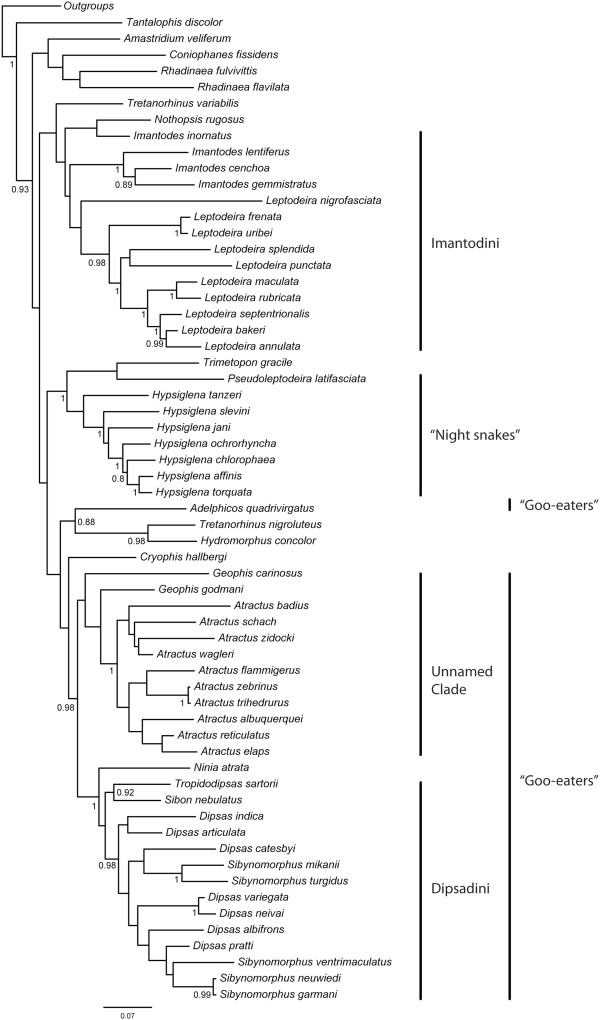
**Bayesian 50% majority-rule consensus tree of Dipsadinae.** Numbers at nodes show Bayesian posterior probability support values (see text for details).

Both ML (-lnL = -65357.67; Figure 14) and BI trees (majority rule consensus of 8500 trees after the burn-in; Figure 15) yield very similar results, differing only in the position of *Tretanorhinus variabilis*, *Trimetopon gracilis*, *Nothopsis rugosus*, and in the affinities between some species within the genera *Atractus* and *Dipsas* (see Additional file 3 and Additional file 4 for the complete tree topologies, including all outgroup taxa). We retrieved only 20 well-supported clades (i.e., BML and BPP frequencies of 70% and 0.8 or more, respectively), which correspond to only 33% of all possible clades. Most higher and lower-level interrelationships are not strongly supported, with the notable exception of the subfamily Dipsadinae (Figures 14, 15). Both ML and BI analyses recovered a well-supported Dipsadinae with a BML of 87% and a BPP of 1.0. *Tantalophis discolor* is positioned as the sister group of all other dipsadines [32], with low BML (<70%), but high BPP (0.93), and *Amastridium veliferum*, *Coniophanes fissidens*, and the genus *Rhadinaea* form a poorly supported clade (BMF < 70% and BPP < 90%) that represents the sister group of the remaining dipsadines. Within that clade, *Coniophanes fissidens* is retrieved as the sister group of the genus *Rhadinaea*, represented in this analysis by *R. flavilata* and *R. fulvivittis*. These two clades are poorly supported in both analyses (BML <70% and BPP <90%).

Both analyses recovered a poorly supported clade (BML <70%; BPP <0.8) grouping the remaining dipsadines distributed in five larger assemblages: 1) “Clade A”, formed by the genera *Nothopsis*, *Imantodes*, and *Leptodeira*; 2) “Clade B”, composed by *Trimetopon* and the “nightsnakes” genera *Hypsiglena* and *Pseudoleptodeira*; 3) “Clade C”, formed by *Adelphicos quadrivirgatus*, *Hydromorphus concolor*, and *Tretanorhinus nigroluteus*; 4) “Clade D”, composed by the goo-eaters *Geophis* and *Atractus*; 5) and “Clade E”, including *Ninia* and the snail-eating snakes *Tropidodipsas*, *Dipsas*, *Sibon*, and *Sibynomorphus*. Clades A, B, and D were retrieved with very low BML and BPP supports (<70%/<0.8), while Clades C and E received low to moderate BML (<70% and 70%, respectively) but high BPP supports (0.88 and 1.0, respectively). The genera *Tretanorhinus*, *Imantodes, Geophis*, *Dipsas*, and *Sibynomorphus* are not non-monophyletic in both ML and BI analyses, and *Hypsiglena* appears as non-monophyletic in the ML analysis. The genera *Rhadinaea*, *Leptodeira*, and *Atractus* are recovered as monophyletic in both analyses, but only *Atractus* receives moderate BML and high BPP support values (72%/1.0).

Within Clade A, *Nothopsis rugosus* is recovered as the sister group of a monophyletic tribe Imantodini (*sensu* Myers [33]) in the ML analysis, whereas in the BI analysis *Nothopsis* appears nested within the latter as the sister group of *Imantodes inornatus*. The genus *Leptodeira* is recovered as monophyletic, albeit with weak support. Similarly, within Clade B, *Trimetopon gracile* is found nested inside the night-snake genus *Hypsiglena* in the ML analysis, whereas in the BI analysis it clusters with *Pseudoleptodeira nigrofasciata* as the sister group of a monophyletic *Hypsiglena*.

*Tretanorhinus variabilis* clusters as the sister group of Clade A in the BI analysis whereas in the ML analysis it is placed as the sister group of a weakly supported clade formed by Clades B, C, D, E, and *Cryophis*.

Surprisingly, Cadle and Greene’s [18] goo-eating snakes are recovered as a polyphyletic assemblage in both analyses, with *Adelphicos quadrivirgatus* included in Clade C as the sister group of *Hydromorphus concolor* and *Tretanorhinus nigroluteus*, whereas the remaining goo-eating genera (Clades D and E) cluster together forming a clade that shows low BML support (<70%) but high BPP support (0.98). However, although our molecular tree points to a polyphyletic goo-eating assemblage, weak support for the nodes separating *Adelphicos* from the other goo-eaters and the expressive amount of morphological traits shared by both groups suggest caution interpreting these results. Within Clade C, *H. concolor* and *T. nigroluteus* form a monophyletic group of Central American aquatic snakes [29] strongly supported by both BML and BPP values (100/0.88). Clade C appears in both analyses as the sister group of the weakly supported clade formed by *Cryophis* and Clades D and E.

*Cryophis hallbergi* is recovered in both analyses as the sister group of a clade formed by the remaining goo-eating genera *Ninia*, *Atractus*, *Geophis*, *Dipsas*, *Sibynomorphus*, *Sibon*, and *Tropidodipsas*, albeit with weak support (<70%/<0.8). The latter clade is recovered with low BML (<70%) but high BPP (0.98) support values. Within that clade, very few nodes enjoy high support values and each probabilistic approach obtained a unique topology. However, some relationships are corroborated by both analyses. As previously shown by Grazziotin el al. [30], the goo-eating genera *Ninia*, *Atractus*, *Geophis*, *Dipsas*, *Sibynomorphus*, *Sibon*, and *Tropidodipsas* form two monophyletic assemblages: Clade D, composed by the genera *Geophis* and *Atractus* with low support values (<70%/<0.8), and Clade E composed by *Ninia*, *Dipsas*, *Sibynomorphus*, *Sibon*, and *Tropidodipsas* with moderate to high support values (70%/1.0). In both analyses, monophyly of *Geophis* is not recovered, with *Geophis godmani* and *G. carinosus* positioned as successive sister groups of *Atractus*. Monophyly of *Atractus* is retrieved with moderate to high support values (72%/1.0), although the relationship among the species of the genus received low support in both analyses. *Ninia atrata* is positioned as the sister group of Dipsadini, with the latter receiving surprisingly low support (<70%/<0.8). Within the tribe Dipsadini, *Sibon nebulatus* and *Tropidodipsas sartorii* form a moderately well supported clade (70%/0.92) that is retrieved as the sister group of a clade composed by *Dipsas* and *Sibynomorphus*. The latter clade is obtained in both analyses, with low BML (<70%) but high BPP (0.98) support values, and recovers both *Dipsas* and *Sibynomorphus* as paraphyletic in respect to each other. Within that paraphyletic assemblage, the following well-supported clades are present in both analyses: 1) *Sibynomorphus turgidus* and *S. mikanii* (78%/1.0), 2) *Dipsas neivai* and *D. variegata* (99%/1.0), 3) *S. garmani* and *S. neuwiedi* (100%/0.99).

## Discussion

The subfamily Dipsadinae is a well-corroborated monophyletic group of Neotropical snakes that has been recently redefined on the basis of both molecular and morphological evidence [12]. However, affinities among dipsadine genera are still largely unknown [30]. Some authors suggested monophyletic groups within this lineage, such as the “niniiforms” [34], the *Leptodeira*-*Eridiphas*, and the *Sibon*-*Geophis* clades [35]. Recently, Mulcahy [32] provided molecular evidence for a clade including the nightsnakes *Eridiphas*, *Hypsiglena*, and *Pseudoleptodeira*, and another clade containing the genera *Leptodeira* and *Imantodes*. Mulcahy’s clade of nighstsnakes is recovered in our phylogenetic analysis with the genus *Trimetopon* nested within, although with low support (Figures 14 and 15).

According to Savitzky [34], the “niniiform” clade includes the genera *Amastridium*, *Chersodromus*, *Diaphorolepis*, *Emmochliophis*, *Ninia*, *Nothopsis*, *Synophis*, and *Xenopholis*. Recent molecular analyses pointed out the polyphyletic nature of niniiforms, with at least *Ninia*, *Xenopholis* and *Nothopsis* nesting distantly from each other within the dipsadid radiation [12,29-31]. Cadle and Greene [18] were the first to explicitly suggest a close affinity of *Ninia* with the tribe Dipsadini, by recognizing a putative clade of seven Central American xenodontine genera of goo-eating snakes that feed exclusively on soft-bodied invertebrates. Interestingly, recent phylogenetic analyses have shown *Ninia* invariably nested within the Tribe Dipsadini, suggesting a paraphyletic condition for the tribe [12,29,30]. However, our analysis retrieved a monophyletic Dipsadini, with *Ninia* positioned outside the latter clade. This result is congruent with the morphological evidence at hand, since *Ninia* lacks the specializations shown by Dipsadini although most species are known to feed mainly on slugs [18] (Additional file 1).

Surprisingly, Cadle and Greene’s [18] goo-eating snakes were recovered as a polyphyletic assemblage in our analysis, with the cryptozoic *Adelphicos* clustering outside the clade formed by the remaining goo-eaters (i.e., *Atractus*, *Geophis*, *Ninia*, *Dipsas*, *Sibon*, *Tropidodipsas*, and *Sibynomorphus*), as the sister group of *Hydromorphus* and *Tretanorhinus* (Clade C in Figure 14). The latter two genera form a strongly supported clade of Central American aquatic snakes [29]. Pyron et al. [29], who included *Adelphicos* for the first time in a molecular phylogenetic analysis, found the same clade formed by *Adelphicos*, *Hydromorphus*, and *Tretanorhinus*, but did not comment on this unexpected result. Our larger sample of Dipsadines failed to support a phylogenetic affinity of *Adelphicos* with the other cryptozoic goo-eating snakes traditionally associated with it (*Atractus* and *Geophis*), suggesting that their specialized feeding habits and morphology evolved independently within Dipsadinae (Figures 14 and 15).

Our results also suggest that the loss of grooved enlarged maxillary teeth and loreal scales, pointed out as evidence in support of the monophyly of the goo-eaters [20], should also be considered homoplastic in *Adelphicos*. However, although our molecular tree points to a polyphyletic goo-eating assemblage, weak support values for the nodes separating *Adelphicos* from the other goo-eaters and the expressive amount of morphological traits shared by both groups indicate that these results are still tentative.

The remaining goo-eating snakes form a moderately supported clade (Figures 14, 15) with low BML but high BPP support values. This monophyletic component, numbered 1 in Figure 14, includes the genera *Geophis* and *Atractus*, on the one hand, and *Ninia*, *Tropidodipsas*, *Sibon*, *Dipsas*, and *Sibynomorphus*, on the other. Both monophyletic components are described as Clades D and E in our Results, and are depicted in green and blue in Figure 14, respectively. While Clade D is formed by cryptozoic species that feed mainly on earthworms, Clade E (including *Ninia*) is essentially terrestrial or arboreal and known to be mainly molluscivorous (Additional file 1).

However, with the exception of *Sibynomorphus*, there have been reports of non-molluscan prey being extensively consumed in captivity or found in stomach contents and fecal samples of species from the remaining four genera in Clade E [36]. Non-molluscan prey items comprise earthworms (in *Dipsas bucephala*, *D. elegans, Ninia sebae, Sibon annulatus, S. argus, S. faciata, S. longifrenis, Tropidodipsas fisheri, T. philippii*), arthropod remains (in *Dipsas catesbyi*, *D. indica*)*,* leeches (in *Ninia sebae*), amphibian eggs (*Sibon longifrenis* and *S. argus*), and Amphisbaenidae (*Ninia sebae*) (Additional file 1). On the other hand, genera in Clade D seem to feed mainly on earthworms (Additional file 1). Nonetheless, some authors also described the presence of arthropod remains in the stomach content of *Geophis incomptus* (and there are indications that this seems to be a common item in several species of *Atractus*), acari (in *Atractus latifrons* and *A. torquatus*), slugs (in *Atractus carrioni, G. nigrocinctus*, and *G. pyburni*), leeches (in *G. nasalis*), and vertebrate remains such as lizards scales (in *A. pantostictus*) (Additional file 1).

Although the large number of specializations found in snail-eating snakes associated with molluscivory points to a single acquisition from a common ancestor [37], a closer inspection of the anatomy of these snakes suggests a more complex evolutionary scenario. In the phylogenetic tree depicted in Figure 14, three arrows, numbered from 1 to 3, indicate the nodes in which key evolutionary novelties arose within the goo-eating radiation and led to the highly specialized protein-secreting delivery system described herein in snail-eating snakes. Some of these novelties also seem to have appeared homoplastically in the cryptozoic genera *Adelphicos* and *Geophis* (arrows with an asterisk in their numbers in Figure 14) [17].

According to our dissections and based on the phylogenetic hypothesis in Figure 14, a fully individualized LAO that attaches ventrally to an enlarged and partially seromucous infralabial gland evolved independently in the genus *Adelphicos* (Arrow 1*) and in the common ancestor of the remaining goo-eating snakes, indicated by Arrow 1 in Figure 14, allowing these snakes to secrete and discharge mainly protein secretions into their mouth and prey. The clade Dipsadini, shown by Arrow 2 in Figure 14, is characterized by an hypertrophied LAO and an extensively folded and loose epithelial tissue covering the floor of the mouth, two synapomorphies that confer more flexibility and strength to mandibular movements. Arrow 3 corresponds to the node of the common ancestor of the genera *Dipsas* and *Sibynomorphus*, in which evolved a divided infralabial gland with a reduced il1 and a distinct, well developed il2 that discharges its protein secretion through a single large duct opening in the epithelium of the mouth floor at the level of the intermandibular raphe, an extended LAO that inserts via an aponeurotic tendon on the lateral surface of the tip of the dentary, and a heavily folded epithelium that accommodates the large LAO laterally to the dentary. Two distinct infralabial portions (il1 and il2) also evolved independently in the genus *Geophis* as depicted in Figure 14 (Arrow 3*). However, the condition in *Geophis* shows several important differences from the one described in *Sibynomorpus* and *Dipsas*, the more important ones being that the larger medial duct in the il2 of *Geophis* represents a real lumen that accumulates secretion and the muscle compressing the il2 corresponds to the muscle *adductor mandibulae externus medialis pars posterior* (AMEM, *sensu* Zaher [28]) instead of the IPP or LAO [17]. The paraphyletic condition of *Geophis* shown in our phylogenetic analysis may suggest that the divided condition of the infralabial gland and specializations of the il2 in that genus were secondarily lost in *Atractus*. However, we suspect that a better sampling of *Geophis* will likely alter this result, and prefer to view any conclusion regarding this group as premature.

## Conclusions

Although chemical properties and some anatomical aspects of the secretion delivery mechanism in Dipsadini could be inferred from the histological, morphological, and behavioral data, the exact role of the Dipsadini infralabial secretion and its use during predation in molluscs and other invertebrates is still largely unknown. Several authors hypothesized that toxins secreted by the infralabial glands of Dipsadini are probably used in some envenomation function or in assisting in the detachment of the snails from their shells [10,14,38,39]. However, as shown by Sazima [39] and here (video provided as Additional file 5), *Dipsas* and *Sibynomorphus* always extract the snail through a sudden strike followed by a sequence of fast alternating insertions of the mandible inside the shell that are meant to rapidly extract the snail and ingest it in the same sequence ([39]; Additional file 5). Such burst of mandibular movement results in a fast mechanical extraction that does not seem to depend on a chemical reaction of any kind. Similarly, observations made with species of the cryptozoic genus *Atractus* have shown that these snakes capture and ingest their prey through a sequence of fast, alternating movements of their mandible and do not seem to depend on any chemical aid from the protein secretion of the infralabial glands to subdue their prey.

As in *Geophis*[17], the presence of a seromucous il2 that is not functionally associated to a specialized tooth row but rather opens loosely in the epithelium of the mouth suggests a function that is likely to be directed to mucus control and prey ingestion rather than prey envenomation. Such function may have evolved associated to a seromucous condition of the infralabial gland il1 in the common ancestor of goo-eating snakes as a system to control unusual flow of mucus and assist in the ingestion of elongate, flexible and highly viscous preys (Figure 14). Evolution of a secretory system specialized in mucus control and prey ingestion, instead of simple prey envenomation, is here thought to represent a key element for the success of goo-eating snakes in the Neotropics.

## Methods

We used throughout the text the term “snail-eating snakes” to refer to the tribe Dipsadini that comprises the genera *Dipsas*, *Sibon*, *Sibynomorphus*, *Plesiodipsas*, and *Tropidodipsas*, and the term “goo-eating snakes” to refer to the larger group that comprises the snail-eating snakes and the genera *Adelphicos*, *Atractus*, *Chapinophis*, *Chersodromus*, *Geophis*, *Ninia*, and *Omoadiphas*[18,20]. The term “goo-eating snakes” will be used only for convenience since it appears to represent a paraphyletic lineage. Also, monophyly of *Tropidodipsas* is still controversial [40]. However, we followed Wallach [41] that resurrected *Tropidodipsas* to accommodate five species (*annulifera*, *fasciata*, *philippii*, *sartorii*, and *zweifeli*) previously referred to *Sibon*. We also recognize herein the subfamily Dipsadinae as defined by Zaher et al. [12].

### Morphological analyses

Representatives from four of the five known genera of Dipsadini were analyzed. Only *Plesiodipsas* was not available for dissection. However, Harvey et al. [22] provided descriptions of the head muscles and glands of the species. We dissected the head of 16 species of *Dipsas*, five *Sibon*, eight *Sibynomorphus*, and two *Tropidodipsas*, totalling 31 species of Dipsadini (see Additional file 2). Additionally to the “snail-eating” taxa, we also dissected 32 species of the goo-eating genera *Adelphicos*, *Atractus*, *Chersodromus*, *Geophis*, and *Ninia*, as well as 31 representatives of 29 additional genera of Dipsadinae (see Additional file 2).

All dissections and drawings were performed under a dissecting microscope Olympus SZX 12 equipped with a *camera lucida*. Specimens dissected in this study belong to the following collections: American Museum of Natural History, New York (AMNH); Instituto Butantan, São Paulo (IBSP); Museu Ecuatoriano de Ciencias Naturales, Quito (MECN); Museu Paraense Emílio Goeldi, Belém (MPEG); Museu de Zoologia da Universidade de São Paulo (MZUSP); Museum of Natural History, University of Kansas, Lawrence (KU); Museum of Natural Science, Louisiana State University, Baton Rouge (LSUMZ); National Museum of Natural History, Washington (USNM); Royal Ontario Museum (ROM).

Studies on the superficial soft tissue morphology of the mouth in snakes are scarce and have been directed to the palate rather than the floor of the mouth. McDowell [42] and Groombridge [43] offered some information on the soft tissue anatomy of the floor of the mouth of snakes, and we follow here their terminology. Glandular terminology follows Taub [13], Kochva [3], and Underwood [8]. Terminology for the muscles of the intermandibular region of snakes follows Langebartel [44] and Groombridge [43]. Terminology for the *adductores externi* muscles is still in dispute among authors [28,45,46]. We follow here the arrangement suggested by Zaher [28].

### Molecular phylogenetic analysis

Our data matrix was composed by 87 terminal taxa and 501 sequences downloaded from GenBank for five mitochondrial (12S, 16S, cytb, nd2, nd4) and six nuclear genes (bdnf, c-mos, jun, nt3, rag1, rag2) (see Additional file 6 for Genbank accession numbers). When multiple sequences were available in GenBank for a given taxon, the most complete sequence was selected for inclusion. We also produced 75 additional sequences aiming to improve the completeness of our matrix. We used 26 outgroup terminals (19 Xenodontinae, three Carphophiinae, and three Natricidae). Sixty-one Dipsadinae terminals composed the ingroup, representing an increase of 25 species in respect to the taxon sampling used by Pyron et al. [29] and 31 species to the one used by Grazziotin et al. [30].

Primers and PCR protocols for partial amplification of genes 12S, 16S, cytb, bdnf, and c-mos were those described in Grazziotin et al. [30]. We used the primers and protocols described in Vidal and Hedges [47], Noonan and Chippindale [48], and Chiari et al. [49] to amplify fragments for the nuclear genes jun, nt3, and rag1, respectively. PCRs were purified with shrimp alkaline phosphatase and exonuclease I (GE Healthcare, Piscataway, NJ, USA) and sequences were processed using the DYEnamic ET Dye Terminator Cycle Sequencing Kit in a MegaBACE 1000 automated sequencer (GE Healthcare) following the manufacturer’s protocols. Both strands were sequenced for all fragments and sequences were assembled using Geneious 5.5 [50].

The multiple sequence alignment process implemented in MAFFT [51] was applied for the rRNA sequences using the iterative refinement method implemented in the E-INS-I algorithm [52]. Otherwise, all sequences for the coding genes were translated to amino acids and aligned based on the Gonnet series matrix implemented in Clustal X [53], and subsequently retro-translated to nucleotides. We concatenated the rRNAs with the retro-aligned coding genes, totalizing 9169 bps. All gaps were coded as missing data.

The concatenated matrix was analyzed by maximum likelihood (ML) and Bayesian inference (BI). We followed Grazziotin et al. [30] and split our matrix to allow the use of different model parameters for each codon position for the coding genes, and for each rRNA sequence. We carried out the ML analysis using RAxML 7.2.8 [54]. The GTRGAMMA model was used for all partitions, as recommended in the program documentation. Forty RAS were built and the trees were swapped using LSR algorithm. To access the bootstrap frequencies for the ML analysis (BML), one thousand pseudoreplications of non-parametric bootstrap were performed using the Cluster hosted at the Laboratório de Alto Desempenho – Pontifícia Universidade Católica do Rio Grande do Sul (LAD-PUCRS). We used MrBayes 3.1.2 [55] to implement BI analyses. Two independent runs with 11 million generations for four chains with a temperature of 0.05 were performed, sampling each 1000^th^ generation. The prior probability densities for substitution rates and for stationary nucleotide frequencies of the rate matrix were selected as suggested by the MrModeltest analysis. A uniform prior was set for topology and the default unconstrained exponential prior was set for branch length parameter. Only the topology and branch lengths were treated as linked parameters among partitions. The log likelihood trace was accessed using Tracer v1.5 [56] and the cutoff for the burn-in was determined as the point at which the trace became stationary. We accessed the average standard deviation of split frequencies to assure the convergence between different MrBayes runs and the Effective Sampling Sizes (ESS) for each parameter were inspected using the program Tracer. A 50% majority-rule consensus tree was constructed using the software TreeAnnotator v.1.5.4 [57]. Frequency of nodal resolution for each clade was termed a Bayesian Posterior Probability (BPP).

### Histology and histochemistry

Histological procedures were performed in 28 adult individuals belonging to the snail-eating genera *Dipsas*, *Sibon*, *Sibynomorphus*, *Tropidodipsas* and the remaining goo-eating genera *Atractus*, *Geophis*, *Ninia*, and *Chersodromus*. We also performed histological sections in seven individuals from the dipsadine genera *Coniophanes*, *Hypsiglena*, *Imantodes*, *Leptodeira*, and *Urotheca* (see Additional file 2). Histological sections were performed on previously fixed specimens housed in several scientific collections. Heads were skinned from the nostril to the neck and removed from the specimens at the level of the first cervical vertebra. Specimens and their head skin were thus returned to their jar in the collection. Heads from cientific collections were post-fixed with Bouin and submitted to decalcification and serial sections in the same way as live individuals (see below).

Additionally, live adult individuals of *Dipsas neivai*, *D. indica*, *D. albifrons*, *Sibynomorphus mikanii*, and *S. neuwiedi* were used in this work for more accurate histological procedures (see Additional file 2). Live specimens were provided by the Laboratório de Herpetologia do Instituto Butantan. They were euthanized through an intraperitoneal injection of sodium thiopental (30 mg/Kg). We removed either the complete head or only the infralabial glands of euthanized specimens for architectural analysis of the glands and associated duct systems and muscles. All specimens were preserved in formalin and deposited in the herpetological collections of the Instituto Butantan and Museu de Zoologia da Universidade de São Paulo.

The heads were fixed in Bouin fixative for 24 hours and posteriorly submitted to decalcification in 4.13% aqueous EDTA, pH 7.2, renewed every other three days, and kept in constant stirring for 60 days. The heads were then sagittally divided in two halves, dehydrated in ethanol, embedded in paraffin, and submitted to serial, sagittal or horizontal sectioning. Sections of 7 μm were obtained in a Microm HM 340 E microtome with disposable steel blades. All sections were submitted to hematoxylin-eosin (HE) staining for general study of the tissues, and to Mallory trichrome staining [58] for the specific identification of collagen and muscular fibres and epithelia.

Dissected glands were fixed during 24 hours in 4% paraformaldehyde in PBS 0.1 M, pH 7.2, dehydrated in ethanol and embedded in historesin (glycol methacrylate, Leica, Nussloch/Heidelberg, Baden-Württemberg, Germany). Sections of 2 μm were obtained in the same Microm HM 340 E microtome mounted with glass knives. Some sections were stained with toluidine blue-fuchsin [59] for a general view of the glandular condition. The remaining historesin sections were subjected to the following histochemical staining procedures [60]: periodic acid-Schiff (PAS), alcian blue pH 2.5 and combined PAS and alcian blue pH 2.5 [61,62] for the identification of neutral (PAS) and acid (alcian blue) mucosubstances, and bromophenol blue for the identification of proteins.

Photomicrographs were obtained with an Olympus BX51 microscope and an Olympus SZ stereomicroscope (Olympus, Tokyo, Japan) equipped with a digital camera and with the software Image-Pro Express (MediaCybernetics, Maryland, USA).

### Availability of supporting data

The matrix is also deposited in the treeBase data repository (http://treebase.org/treebase-web/home.html), available through http://treebase.org/treebase-web/search/study/summary.html?id=15015.

Referee’s link: http://purl.org/phylo/treebase/phylows/study/TB2:S15015?x-access-code=a7076770a44cfb15c2b2fad39178b5da&format=html.

## Competing interests

The authors declare that they have no competing interests.

## Authors’ contributions

HZ conceived the research. HZ, LO, ALP designed the research. HZ, LO, FGG, MC, CJ, MMA, ALP analyzed the data. HZ, LO, FGG, MC, CJ, MMA, ALP wrote the paper. HZ, LO, FGG, ALP prepared the figures. All authors read and approved the final manuscript.

## Supplementary Material

Additional file 1 A list of prey items recovered from the literature on dipsadine snakes. Click here for file

Additional file 2 A list of specimens used in this study. Click here for file

Additional file 3 **Tree estimated from a Maximum Likelihood analysis of 11 concatenated genes using RAxML 7.2.8.** All outgroups are shown in the tree. Bootstrap values greater than 70% are given above each node. Click here for file

Additional file 4 **Fifty percent Majority-rule consensus tree estimated from a Bayesian analysis of 11 concatenated genes using MrBayes 3.1.2.** All outgroups are shown in the tree. Bayesian Posterior Probability frequencies greater than 0.80 are given above each node. Click here for file

Additional file 5 **A movie showing the predation sequence of an adult specimen of *Dipsas albifrons *on a snail of the genus *Bradybaena, *filmed in captivity at the biotherium of the Laboratório de Biologia Celular of the Instituto Butantan.** Specimen was collected in Jaraguá do Sul, State of Santa Catarina, Brazil (body length = 525 mm; tail length = 183 mm). Click here for file

Additional file 6 A List of DNA sequences used in this study, with GenBank accession numbers. Click here for file

## References

[B1] Smith M Bellairs Ad A The head glands of snakes, with remarks on the evolution of the parotid gland and teeth of the Opisthoglypha J Linn Soc Zool 1947 41 351 370 10.1111/j.1096-3642.1940.tb02079.x

[B2] Underwood G Thorpe RS, Wüster W, Malhota A An overview of venomous snake evolution Venomous snakes: ecology, evolution and snakebite. Symposia of the Zoological Society of London 70 1997 Oxford: Clarendon Press 1 13

[B3] Kochva E Gans C, Gans KA Oral glands of the Reptilia Biology of the Reptilia, Volume 8 1978 New York: Academic Press 43 161

[B4] Vidal N Colubroid systematics: evidence for an early appearance of the venom apparatus followed by extensive evolutionary tinkering J Toxicol Toxin Rev 2002 21 21 41 10.1081/TXR-120004740

[B5] Kardong KV Colubrid snakes and Duvernoy’s “venom” glands J Toxicol Toxin Rev 2002 21 1 19 10.1081/TXR-120004739

[B6] Fry BG Wüster W Assembling an arsenal: origin and evolution of the snakes venom proteome inferred from phylogenetic analysis of toxin sequence Mol Biol Evol 2004 21 870 883 10.1093/molbev/msh091 15014162

[B7] Fry BG Vidal N Norman JA Vonk FJ Scheib H Ramjan SFR Kuruppu S Fung K Hedges SB Richardson MK Hodgson WC Ignjatovic V Summerhayes R Kochva E Early evolution of the venom system in lizards and snakes Nature 2006 439 584 589 10.1038/nature04328 16292255

[B8] Underwood G On the rictal structures of some snakes Herpetologica 2002 58 1 17 10.1655/0018-0831(2002)058[0001:OTRSOS]2.0.CO;2

[B9] Deufel A Cundall D Functional plasticity of the venom delivery system in snakes with focus on the poststrike prey release behavior Zool Anz 2006 245 249 267 10.1016/j.jcz.2006.07.002

[B10] Oliveira L Jared C Prudente ALC Zaher H Antoniazzi MM Oral glands in dipsadine “goo-eater” snakes: morphology and histochemistry of the infralabial glands in *Atractus reticulatus*, *Dipsas indica*, and *Sibynomorphus mikanii* Toxicon 2008 51 898 913 10.1016/j.toxicon.2007.12.021 18262581

[B11] Saint Girons H Societé Herpétologique de France Évolution de la fonction venimeuse chez les reptiles Comptes-Rendus du Colloque Serpents, Venins, envenimations: 2 July 1987; Lyon 1989 Lyon: Fondation Marcel Mérieux 9 22

[B12] Zaher H Grazziotin FG Cadle JE Murphy RW Moura-Leite JC Bonatto SL Molecular phylogeny of advanced snakes (Serpentes, Caenophidia) with an emphasis on South American xenodontines: a revised classification and descriptions of new taxa Pap Avulsos Zool 2009 49 1 153

[B13] Taub AM Ophidian cephalic glands J Morphol 1966 118 529 542 10.1002/jmor.1051180406 5956246

[B14] Zaher H A musculatura associada à glândula infralabial de *Dipsas neivai*: um novo sistema de inoculação de veneno relacionado à malacofagia (Serpentes, Dipsadinae) [abstract] Resumos do 21° Congresso Brasileiro de Zoologia: 5–9 February 1996 1996 Porto Alegre: Rio Grande do Sul: Sociedade Brasileira de Zoologia 201

[B15] Haas G Über die Schädelmechanik und die Kiefermuskulatur einiger Proteroglypha Zool Jahrb Anat 1930 52 347 404

[B16] Savitzky AH Coadapted character complexes among snakes: fossoriality, piscivory, and durophagy Am Zool 1983 23 397 409

[B17] Oliveira L Prudente ALC Zaher H Unusual labial glands in snakes of the genus *Geophis* Wagler, 1830 (Serpentes: Dipsadinae) J Morphol 2014 275 87 99 10.1002/jmor.20199 24127255

[B18] Cadle JE Greene HW Ricklefs RE, Schluter D Phylogenetic Patterns, Biogeography, and the Ecological Structure of Neotropical Snake Assemblages Species Diversity in Ecological Communities: Historical and Geographical Perspectives 1993 Chicago: University of Chicago Press 281 293

[B19] Passos P Fernandes R Porto M Geographical variation and taxonomy of the snail-eating snake *Dipsas albifrons* (Sauvage, 1884), with comments on the systematic status of *Dipsas albifrons cavalheiroi* Hoge, 1950 (Serpentes: Colubridae: Dipsadinae) Zootaxa 2005 1013 19 34

[B20] Campbell JA Smith EN A new genus and species of colubrid snake from the Sierra de Las Minas of Guatemala Herpetologica 1998 54 207 220

[B21] Köhler GLD Wilson LD McCranie JR A new genus and species of colubrid snake from the Sierra de Omoa of northwestern Honduras (Reptilia, Squamata: Colubridae) Senckenb Biol 2001 81 269 176

[B22] Harvey MB Fuenmayor GR Portilla JRC Rueda-Almonacid JV Systematics of the enigmatic dipsadine snake *Tropidodipsas perijanensis* Alemán (Serpentes: Colubridae) and review of morphological characters of Dipsadini Herpetol Monogr 2008 22 106 132

[B23] McCranie JR A description of the first male of the colubrid snake genus *Omoadiphas*, with an expanded definition of the genus Caribb J Sci 2006 42 271 272

[B24] Peters JA The snakes of the subfamily Dipsadinae Misc Publ Mus Zool Univ Mich 1960 114 1 224

[B25] Haas G Über die morphologie der Kiefermuskulatur und die Schädelmechanik einiger Schlangen Zool Jb Anat 1931 54 333 416

[B26] Haas G A note on the origin of solenoglyph snakes Copeia 1938 1938 73 78 10.2307/1435694

[B27] Scott NS Jr The colubrid snake, *Tropidodipsas annulifera*, with reference to the status of *Geatractus*, *Exelencophis*, *Chersodromus annulatus*, and *Tropidodipsas malacodryas* Copeia 1967 1967 280 287 10.2307/1442115

[B28] Zaher H Comments on the evolution of the jaw adductor musculature of snakes Zool J Linn Soc 1994 111 339 384 10.1111/j.1096-3642.1994.tb01488.x

[B29] Pyron RA Burbrink FT Colli GR de Oca ANM Vitt LJ Kuczynski CA Wiens JJ The phylogeny of advanced snakes (Colubroidea), with discovery of a new subfamily and comparison of support methods for likelihood trees Mol Phylogenet Evol 2011 58 329 342 10.1016/j.ympev.2010.11.006 21074626

[B30] Grazziotin FG Zaher H Murphy RW Scrocchi G Benavides MA Zhang Y-P Bonatto SL Molecular phylogeny of the new world dipsadidae (Serpentes: Colubroidea): a reappraisal Cladistics 2012 28 437 459 10.1111/j.1096-0031.2012.00393.x 34836446

[B31] Vidal N Dewynter M Gower DJ Dissecting the major American snake radiation: a molecular phylogeny of the Dipsadidae Bonaparte (Serpentes, Caenophidia) C R Biol 2010 333 48 55 10.1016/j.crvi.2009.11.003 20176336

[B32] Mulcahy DG Molecular systematics of Neotropical cat-eyed snakes: a test of the monophyly of Leptodeirini (Colubridae: Dipsadinae) with implications for character evolution and biogeography Biol J Linn Soc Lond 2007 92 483 500 10.1111/j.1095-8312.2007.00855.x

[B33] Myers CW A new genus and new tribe for *Enicognathus melanauchen* Jan, 1863, a neglected South American snake (Colubridae: Xenodontinae), with taxonomic notes on some Dipsadinae Amer Mus Novitates 2012 3715 1 33

[B34] Savitzky AH The origin of the New World proteroglyphous snake and its bearing on the study of venom delivery systems in snake PhD Thesis 1974 University of Kansas: Systematics and Ecology Department

[B35] Cadle JE Molecular systematic of neotropical xenodontine snakes: II: central American xenodontines Herpetologica 1984 40 21 30

[B36] Ray JM Montgomery CE Mahon HK Savitzky AH Lips KR Goo-eaters: diets of the neotropical snakes *Dipsas* and *Sibon* in Central Panama Copeia 2012 2012 197 202 10.1643/CH-10-100

[B37] Cadle JE The snake genus *Sibynomorphus* (Colubridae: Dipsadinae: Dipsadini) in Peru and Ecuador, with comments on the systematics of Dipsadini Bull Mus Comp Zool 2007 158 183 284 10.3099/0027-4100(2007)158[183:TSGSCD]2.0.CO;2

[B38] Salomão MG Laporta-Ferreira IL The role of secretions from the supralabial, infralabial, and Duvernoy’s glands of the slug-eating snake *Sibynomorphus mikanii* (Colubridae: Dipsadinae) in the immobilization of molluscan prey J Morphol 1994 28 369 371

[B39] Sazima I Feeding behavior of the snail-eating snake: Dipsas indica J Herpetol 1989 23 464 468 10.2307/1564072

[B40] McCranie JR The snakes of Honduras–Systematic, Distribution, and Conservation, Society for the study of amphibians and reptiles 2011 26 Michigan: Thomson-Shore Inc.

[B41] Wallach V Revalidation of the genus *Tropidodipsas* Günther, with notes on the Dipsadini and Nothopsini (Serpentes: Colubridae) J Herpetol 1995 29 476 481 10.2307/1565006

[B42] McDowell SB Dobzhansky TH, Hecht MK, Steere WC The evolution of the tongue of snakes, and its bearing on snake origins Evolutionary Biology 1972 6 New York: Appleton-Century-Crofts 191 273

[B43] Groombridge BC Variations in morphology of the superficial palate of henophidian snakes and some possible systematic implications J Nat Hist 1979 13 447 475 10.1080/00222937900770361

[B44] Langebartel DA The hyoid and its associated muscles in snakes Ill Biol Monogr 1968 38 1 156

[B45] Rieppel O The trigeminal jaw adductors of primitive snake and their homologies with the lacertilian jaw adductors J Zool 1980 190 447 471

[B46] McDowell SB The architecture of the corner of the mouth of colubroid snakes J Herpetol 1986 20 353 407 10.2307/1564502

[B47] Vidal H Hedges SB The phylogeny of squamate reptiles (lizards, snakes, and amphisbaenians) inferred from nine nuclear protein-coding genes C R Biol 2005 328 1000 1008 10.1016/j.crvi.2005.10.001 16286089

[B48] Noonan BP Chippindale PT Dipersal and vicariance: the complex evolutionary history of boid snakes Mol Phylogenet Evol 2006 40 347 358 10.1016/j.ympev.2006.03.010 16624591

[B49] Chiari Y Vences M Vietes DR Rabemananjara F Bora P Ramilijaona Ravoahangimalala OR Meyer A New evidence for parallel evolution of color patterns in Malagasy poison frogs (*Mantella*) Mol Ecol 2004 13 3763 3774 10.1111/j.1365-294X.2004.02367.x 15548289

[B50] Drummond AJ Ashton B Buxtonb S Cheung M Cooper A Duran C Field M Heled J Kearse M Markowitz S Moir R Stones-Havas S Sturrock S Thierer T Wilson A Geneious v5.4 2011 Available at: http://www.geneious.com/

[B51] Katoh K Misawa K Kuma K Miyata T MAFFT: a novel method for rapid multiple sequence alignment based on fast Fourier transform Nucleic Acids Res 2002 30 3059 3066 10.1093/nar/gkf436 12136088 PMC135756

[B52] Katoh K Kuma K Toh H Miyata T MAFFT version 5: improvement in accuracy of multiple sequence alignment Nucleic Acids Res 2005 33 511 518 10.1093/nar/gki198 15661851 PMC548345

[B53] Thompson JD Gibson TJ Plewniak F Jeanmougin F The clustal windows interface: flexible strategies for multiple sequence alignment aided by quality tools Nucleic Acids Res 1997 25 4876 4882 10.1093/nar/25.24.4876 9396791 PMC147148

[B54] Stamatakis A RAxML-VI-HPC: maximum likelihood-based phylogenetic analyses with thousands of taxa and mixed models Bioinformatics 2006 22 2688 2690 10.1093/bioinformatics/btl446 16928733

[B55] Ronquist F Huelsenbeck JP MrBayes 3: Bayesian phylogenetic inference under mixed models Bioinformatics 2003 19 1572 1574 10.1093/bioinformatics/btg180 12912839

[B56] Rambaut A Drummond AJ Tracer v1.4 2007 Available at http://tree.bio.ed.ac.uk/software/tracer/

[B57] Drummond AJ Rambaut A Beast: Bayesian evolutionary analysis sampling trees BMC Evol Biol 2007 7 214 10.1186/1471-2148-7-214 17996036 PMC2247476

[B58] Junqueira LCU Bignolas G Brentani R Picrosirius staining plus polarization microscopy, a specific method for collagen detection in tissue sections Histochem J 1979 11 447 455 10.1007/BF01002772 91593

[B59] Junqueira LCU Histology revisited-technical improvement promoted by the use of hydrophilic resin embedding Rev Cien Cult 1995 47 92 95

[B60] Brancroft JD Stevens A Theory and practice of histological techniques 1996 4 New York: Churchill Livingstone

[B61] Pearse AG Histochemistry: theoretical and applied, Volume 2 1985 4 Edinburg: Churchill Livingstone

[B62] Kiernan JA Histological and histochemical methods - theory and practice 2001 London: Oxford University Press

