# Supplementary material for: Consuming viscous prey: a novel protein-secreting delivery system in neotropical snail-eating snakes
Source: BMC Evol Biol. 2014 Mar 25;14:58. doi: 10.1186/1471-2148-14-58 (PMC4021269; doi:10.1186/1471-2148-14-58)
Supplement: Additional file 1 — A list of prey items recovered from the literature on dipsadine snakes. [file 1471-2148-14-58-S1.doc]

**BMC Evolutionary Biology**

**Additional File 1**

**Article: Consuming viscous prey: A Novel Protein-Secreting Delivery System in Neotropical Snail-Eating Snakes**

Authors: Hussam Zaher, Leonardo de Oliveira, Felipe G Grazziotin, Michelle Campagner, Carlos Jared, Marta M Antoniazzi, Ana L Prudente

**Prey items listed in the literature for dipsadine snakes**

When available, the number of specimens for each species of snake that were analyzed for stomach contents is given in parenthesis.

| **Species** | **Prey items** | **References** |
| --- | --- | --- |
| *Adelphicos quadrivirgatus* | earthworms (2) | [1] [2] |
| *Amastridium veliferum* | anuran remains (1) | [3] |
| *Atractus alphonsehogei* | earthworms (1) | [4] |
| *Atractus carrioni* | slugs (2) | [5] |
| *Atractus latifrons* | earthworms (1), earthworm chaetae, acari, insect remains (5) | [6] |
| *Atractus major* | earthworms (4), acari, insect remains (2) | [6] [7] |
| *Atractus pantostictus* | earthworms (12) | [8] [9] |
| *Atractus pantostictus* | lizard scales (1) | [9] |
| *Atractus poeppigi* | insect remains (2), earthworm (2), earthworm chaetae (1) | [6] [7] |
| *Atractus reticulatus* | earthworm remains (2) | [8] |
| *Atractus reticulatus* | earthworms (1) | Campagner & Zaher (pers. obs.) |
| *Atractus schach* | earthworm chaetae and insect remains (1) | [6] |
| *Atractus snethlageae* | earthworms chaetae (3), insect remains (2) | [6] [7] |
| *Atractus torquatus* | earthworm (1), earthworm chaetae, acari, insect remains (21) | [6] [7] |
| *Atractus trihedurus* | earthworms | [10] |
| *Atractus zebrinus* | earthworms, leeches | [10] |
| *Chersodromus sp* | unknown | - |
| *Coniophanes fissidens* | anurans (2) | [1] |
| *Coniophanes fissidensis* | salamanders (1), anurans (68), amphibian eggs (9) | [2] |
| *Coniophanes fissidens* | reptile eggs (13), lizards (19), snakes (11), invertebrates (2) | [2] |
| *Cryophis hallbergi* | small vertebrates | [11] |
| *Dipsas albifrons* | snails (1) | [12] |
| *Dipsas brevifacies* | deshelled snail remains (11) | [13] |
| *Dipsas bucephala* | slugs, snails (3), earthworms (1) | Campagner & Zaher (pers. obs.) |
| *Dipas catesbyi* | arthropod remains, snails, slugs | [14] [15] |
| *Dipsas elegans* | earthworms (1) | [5] |
| *Dipsas gaigeae* | snails (2) | [13] [16] |
| *Dipsas indica* | slugs, snails, ants (23) | [14] [17] |
| *Dipsas nicholsoi* | slugs and snails remains (1) | [18] |
| *Dipsas pavonina* | slugs and sails (8); lizard remains (1) | [7] |
| *Dipsas sp* | snails (49), earthworm chaetae (1) | [19] |
| *Dipsas variegata* | snails, slugs | [12] [20] |
| *Eridiphas slevini* | anurans (1), anuran remains (10, lizards (2) | [21] [22][23] |
| *Geophis anocularis* | earthworms (3) | [24] |
| *Geophis bdubius* | earthworms (4) | [24] |
| *Geophis incomptus* | arthropod remains (1) | [25] |
| *Geophis nasalis* | earthworms, leeches (3) | [2] |
| *Geophis nigrocinctus* | slugs (2) | [25] |
| *Geophis pyburni* | earthworms, slug remains (1) | [25] |
| *Imantodes cenchoa* | reptile eggs and lizard (1) | [1] |
| *Imantodes cenchoa* | lizards (11), anurans (2) | [7] [26] |
| *Imantodes gemmistratus* | lizards (6) | [26] |
| *Imantodes lentiferus* | anurans, lizards (1) | [26] |
| *Leptodeira annulata* | anurans (1) | [27] |
| *Leptodeira annulata* | anurans, lizards (47) | [28][29] |
| *Leptodeira bakeri* | lizards (3) | [28] |
| *Leptodeira fissidens* | anurans, lizards (1) | [28] |
| *Leptodeira maculata* | anurans (5) | [28] |
| *Leptodeira nigrofasciata* | lizards (1) | [28] |
| *Leptodeira punctata* | anurans (1) | [28] |
| *Leptodeira septentrionalis* | anuran eggs, anurans, lizards (11) | [28] |
| *Leptodeira splendida* | anuran eggs, anurans, lizards (3) | [28] |
| *Ninia diademata* | snails, slugs (2) | [1] [2] |
| *Ninia sebae* | caecilian (1), earthworms, leeches, slugs, snails, mollusk remains (11) | [1] [2] [30] |
| *Omoadiphas aurula* | unknown | - |
| *Omoadiphas cannula* | unknown | - |
| *Omoadiphas texiguatensis* | unknown | - |
| *Sibon annulatus* | snails (9), earthworm chaetae (7) | [19] |
| *Sibon annulifera* | slugs (2) | [32] |
| *Sibon anthracops* | slugs (3) | [33] |
| *Sibon argus* | mollusk remains (1), earthworms (26), anuran eggs (11), anuran (1) | [19] [34] [35] |
| *Sibon fasciata* | slugs (4), earthworms (1) | [33] |
| *Sibon fischeri* | snails (13), earthworms (8) | [36] |
| *Sibon lamari* | snails (1) | [34] |
| *Sibon longifrenis* | earthworms (2), amphibian eggs (6) | [19] |
| *Sibon nebulatus* | slugs (1), snails | [19] |
| *Sibon sanniolus* | snails | [37] |
| *Sibon sartorii* | slugs (38) | [32] |
| *Sibynomorphus mikanii* | slugs (286) | [8] [10] [38] |
| *Sibynomorphus neuwiedi* | slugs (2), snails (250) | [8] [10] [38] |
| *Sibynomorphus turgidus* | slugs, snails | [39] |
| *Sibynomorphus ventrimaculatus* | slugs, snails (41) | [40] |
| *Tretanorhinus nigroluteus* | fish (1) | [41] |
| *Tretanorhinus variabilis* | fish (1) | [42] |
| *Urotheca decipiens* | anurans, salamanders, lizards | [43] |
| *Urotheca guentheri* | anurans | [44] |

**Supplementary References**

1. Landy MJ, Langebartel DA, Moll EO, Smith HM (1966) A collection of snakes from Volcan Tacana, Chiapas, Mexico. J Ohio Herpetol Soc 5(3): 93-101.

2. Seib RL (1985) Feeding ecology and organization of neotropical snake faunas. Berkeley: University of California.

3. Blaney RM, Blaney PK (1978) Additional specimens on *Amastridium veliferum* Cope (Serpentes: Colubridae) from Chiapas, Mexico. Southwest Nat 23 (4): 692.

4. Cunha OR, Nascimento FP (1983). Ofídios da Amazônia. As espécies de *Atractus* Wagler, 1828 na Amazônia Oriental, Maranhão (Ophidia, Colubridae). Bol Mus Paraense Emilio Goeldi 123: 1-38.

5. Cisnero-Heredia DF (2005) Report of molluscivory in *Atractus carrion* Parker, 1930. Herpetozoa 18: 185.

6. Martins M, Oliveira ME (1993) The snakes of genus *Atractus* Wagler (Reptilia: Squamata: Colubridae) from the Manaus region, Central Amazonia, Brazil. Zool Med Leiden 67.

7. Martins M, Oliveira ME (1998) Natural history of snakes in forests of the Manaus region, Central Amazonia, Brazil. Herpetol Nat Hist 6 (20): 78-150.

8. Barbo FE (2008) Composição, história natural, diversidade e distribuição das serpentes no município de São Paulo, SP. São Paulo: Universidade de São Paulo.

9. Sawaya RJ, Marques OAV, Martins M (2008) Composition and natural history of Cerrado snake assemblage at Itirapina, São Paulo State, Southeastern Brazil. Biota Neotropica 8 (2): 127-149.

10. Marques OAV, Eterovic A, Sazima I. Serpentes da Mata Atlântica. Guia ilustrado para a Serra do Mar. Ribeirão Preto: Editora Holos. 184 pp.

11. Mulcahy DG (2007) Molecular systematics of neotropical cat-eyed snakes: a test of the monophyly of Leptodeirini (Colubridae: Dipsadinae) with implications for character evolution and biogeography. Biol J Linn Soc Lond 92: 483–500.

12. Mertens R (1952) On snail-eating snakes. Copeia 1952: 279.

13. Kofron CP (1982) A review of the mexican snail-eating snakes, *Dipsas brevifacies* and *Dipsas gaigeae*. J Herpetol 16 (3): 270-286.

14. Beebe W (1946) Field notes on the snakes of Kartabo, British Guiana and Caripito, Venezuela Zool 31 (1): 11-52.

15. Bernarde PS, Abe AS (2010) Hábitos alimentares de serpentes em Espigão do Oeste, Rondônia, Brasil. Biota Neotropica 10: 167-173.

16. Harris HS, Simmons RS (1967) Another *Dipsas gaigeae* (Oliver) from Colima, Mexico. Herpetologica 23: 234-235

17. Sazima I (1989) Feeding behavior of the snail-eating snake, *Dipsas indica*. J Herpetol 23 (4): 464-468.

18. Cadle JE, Myers, CW (2003) Systematics of snakes referred to *Dipsas variegata* in Panama and western South America, with revalidation of two species and notes on defensive behaviors in the Dipsadini (Colubridae). Am Mus Novit 3409. 47p.

19. Ray JM, Montgomery CE, Mahon HK, Savitzky AH, Lips KR (2012) Goo-eaters: diets of the neotropical snakes *Dipsas* and *Sibon* in Central Panama. Copeia 2: 197-202.

20. Barrio-Amorós CL, Duellman WE (2009) Herpetofauna de la Sierra de Lema, Estado Bolívar, Venezuela. Bull RAP Eval Ecol 55: 137-155.

21. Grismer l (2002) Amphibians and reptiles of Baja California, including its pacific islands and the islands in the sea of Cortes. Berkeley: University of California. 399p.

22. McPeak RH (2000) Amphibians and Reptiles of Baja California. Monterey: Sea Challengers. 99 p.

23. Mulcahy DG, Archibald MA (2003) Geographic variation in the Baja California nightsnake (*Eridiphas slevini*), with comments on taxonomy and diet. J Herpetol 37 (3): 566-571.

24 Campbell JA, Ford LS, Karges JP (1983) Resurrection of *Geophis anocularis* Dunn with comments of its relationships and natural history. Trans Kansas Acad Sci 86: 38–47.

25. Campbell JA, Murphy JB (1977) A new species of *Geophis* (Reptilia, Serpentes, Colubridae) from the Sierra de Coalcomán, Michoacán, Mexico. J Herpetol 11 (4): 397–403.

26. Myers CW (1982) Blunt-headed vine snakes (*Imantodes*) in Panama, including a new species and other revisionary notes. Am Museum Nov 2738: 1–50.

27. Stuart LC (1948) The Amphibians and Reptiles of Alta Verapaz, Guatemala. Misc Publ Mus Zool Univ Michigan 69: 1-109.

28. Duellman DE (1958) A monographic study of the Colubrid snake genus *Leptodeira*. Bull Am Mus Nat Hist 114 (1): 1-183.

29. Minton SA, Smith HM (1960) A new subspecies of *Coniophanes fissidens* and notes on Central American amphibians and reptiles. Herpetol 16: 103-111.

30. Greene HW (1975) Ecological observations on the red coffee snake, *Ninia sebae*, in southern Veracruz, Mexico. Am Mid Naturalist 93: 478-484.

31. McCranie JR (2011) The Snakes of Honduras – Systematics, Distribution, and Conservation. Contributions to Herpetology. Michigan: Society for the Study of Amphibians and Reptiles, Thomson-Shore. 724p.

32. Kofron CP (1988) Systematics of neotropical gastropod-eating snakes: The *sartorii* group of the genus *Sibon*. Amphibia-Reptilia 9: 145-168.

33. Kofron CP (1987) Systematics of neotropical gastropod-eating snakes: The *fasciata* group of the genus *Sibon*. J Herpetol 21 (3): 210-225.

34. Solórzano A (2002). Una nueva especie de serpiente del genero *Sibon* (Serpentes: Colubridae) de la vertiente del Caribe de Costa Rica. Rev Biol Trop 49: 1111-1120.

35. Ryan MJ, Lips KR. *Sibon argus* (NCN) diet. Herpetol Review 35:278.

36. Kofron CP (1985). Systematics of the neotropical gastropod-eating snake genera *Tropidodipsas* and *Sibon*. J Herpetol 19(1): 84-92.

37. Kofron CP (1983) Female reproductive cycle of the neotropical snail-eating snake *Sibon sanniola* in northern Yucatan, Mexico. Copeia 4: 963-969.

38. Laporta-Ferreira IL, Salomão MG, Sawaya P (1986). Biologia de *Sibynomorphus* (Colubridae: Dipsadinae). Reprodução e hábitos alimentares. Rev Bras Biol 46 (4): 793-799.

39. Melgarejo AR (1980) Comportamiento depredador de *Sibynomorphus turgidus* (COPE) (Serpentes: Dipsadinae). Res I Jornada Ciências Nat Montevidéo 1: 127-128.

40. Oliveira JL (2001) Ecologia de três espécies de dormideira, *Sibynomorphus* (Serpentes: Colubridae). São Paulo: Universidade de São Paulo.

41. Henderson RW, Hoevers LG (1977) The seasonal incidence of snakes at a locality in northern Belize. Copeia 2: 349-355.

42. Schwartz A, Ogren LH (1956). A collection of reptiles and amphibians from Cuba, with descriptions of two new forms. Herpetologica 12(2): 91-110.

43. Montgomery CE, Rodríguez EJG, Ross HL, Jaramillo CJ, Lips KR (2006) *Urotheca decipiens*. Diet. Herpetol Rev 37: 2236.

44. Savage JM (2002) The amphibians and reptiles of Costa Rica: A herpetofauna between two continents, between two seas. Chicago: University of Chicago Press. 934p.
