# Supplementary material for: Consuming viscous prey: a novel protein-secreting delivery system in neotropical snail-eating snakes
Source: BMC Evol Biol. 2014 Mar 25;14:58. doi: 10.1186/1471-2148-14-58 (PMC4021269; doi:10.1186/1471-2148-14-58)
Supplement: Additional file 2 — A list of specimens used in this study. [file 1471-2148-14-58-S2.doc]

**BMC Evolutionary Biology**

**Additional File 2**

**Consuming viscous prey: A Novel Protein-Secreting Delivery System in Neotropical Snail-Eating Snakes**

Hussam Zaher, Leonardo de Oliveira, Felipe G Grazziotin, Michelle Campagner, Carlos Jared, Marta M Antoniazzi, Ana L Prudente

**List of specimens used in this study.**

Acronyms used herein are as follow: American Museum of Natural History, New York (AMNH); Instituto Butantan, São Paulo (IBSP); Museu Ecuatoriano de Ciencias Naturales, Quito (MECN); Museu Paraense Emílio Goeldi, Belém (MPEG); Museu de Zoologia da Universidade de São Paulo (MZUSP); Museum of Natural History, University of Kansas, Lawrence (KU); Museum of Natural Science, Louisiana State University, Baton Rouge (LSUMZ); National Museum of Natural History, Washington (USNM); Royal Ontario Museum (ROM).

**Specimens used in histological and histochemical procedures:**

*Adephicos quadrivirgatum* – USNM 570415, MVZ 169658

*Atractus pantostictus* – MZUSP 15561, 15562

*Atractus reticulatus* – IBSP 77781, 76413

*Atractus zebrinus* – uncatalogued specimen

*Chersodromus liebmanni* – USNM 109920

*Coniophanes fissidens* – USNM 561024

*Dipsas albifrons* – MZUSP 17885, 17233

*Dipsas indica* – IBSP 73451, MZUSP 16695

*Dipsas neivai* – IBSP 74123, MZUSP 14665,15706

*Geophis brachycephalus* – KU 35878

*Geophis nasalis* – MVZ 148267, 148181

*Geophis semidoliatus* – USNM 224839

*Hypsiglena torquata* – MVZ 129353

*Imantodes cenchoa* – MZUSP 15944, 15702

*Leptodeira annulata* – MZUSP 16948, 16958

*Ninia hudsoni* – MZUSP 8344

*Ninia sebae* – MVZ 159410, USNM 109854

*Sibon nebulatus* – MZUSP 9316

*Sibynomorphus mikanii* – MZUSP 17886, 17882

*Sibynomorphus neuwiedi* – MZUSP 17225, 17227

*Tropidodipsas sartorii* – MZUSP 561067

*Urotheca elapoides* – USNM 110765

**Specimens used for muscular and glandular dissections:**

Dipsadini:

*Dipsas* *albifrons* – IBSP 6254, IBSP 55147, MZUSP 70098, MZUSP 70096

*D. alternans* – IBSP 27897, IBSP 32871, IBSP 55951, IBSP 55952, MZUSP 1575, MZUSP 8833; MZUSP 7339

*D. brevifascies* – LSUMZ 33232

*D*. *bucephala* – IBSP 34180, IBSP 49245, IBSP 34275, MZUSP 2460

*D. catesbyi* – KU 121313, IBSP 31346, IBSP 49321

*D. incerta* – IBSP 55951, IBSP 55952

*D. indica* – KU 155508, MZUSP 10126, MECN 119, IBSP 40137, IBSP 52286, IBSP 51073

*D. gracilis* – MZUSP 7705

*D. neivai* – IBSP 51206, IBSP 54935,

*D. oreas* – MZUSP 5710, MECN 3965

*D. pakaraima* – ROM 41234

*D. pavonina* – MPEG 2033

*D. peruana* – MECN 4857

*D. sanctijoannis* – IBSP 7231, IBSP 7222, MZUSP 6353

*D*. *temporalis* – KU 110296, KU 110302

*D. tenuissima* – MZUSP 2049

*D. variegata* – MPEG 12755, MZUSP 8279

*Sibon annulatus* – KU 112480

*S. carri* – LSUMZ 23830

*S.* *dimidiatus* – AMNH 79952, KU 87 467

*S. nebulatus* – AMNH 97068, KU 112474, KU 112476, MZUSP 9668, MZUSP 7828

*S. sanniolus* – KU 70886, KU 157617, LSUMZ 33197

*Sibynomorphus garmani –* IBSP 69222, IBSP 51133

*S. lavillai –* IBSP 68983, IBSP 55374

*S. mikanii* – IBSP 594, IBSP 16361

*S. neuwiedi* – IBSP 55892, IBSP 37523, IBSP 45611, IBSP 55893

*S. petersi* – KU 221727

*S. ventrimaculatus* – IBSP 27871, IBSP 9877, IBSP 17228

*S. vagus* – KU 135180

*S*. *turgidus* – IBSP 46431, IBSP 25193

*S. williamsi* – MECN

*Tropidodipsas fischeri* – KU 63904, KU 63902

*T. sartorii* – KU 157636, KU 157638, AMNH 81940, LSUMZ 23243, MZUSP 8798

Other Dipsadinae:

*Adelphicos veraepacis* – KU 187320, KU 190893

*Amastridium veliferum* – KU 190897

*Atractus albuquerquei* – MPEG 17924

*A. crassicaudatus* – AMNH 67138, IBSP 7210

*A.* *duboisi* – USNM 232583

*A. flammigerus* – KU 123985, KU 125996

*A. guenteri* – IBSP 48889

*A. insipidus* – MPEG 17916

*A. latifrons* – IBSP 43397

*A. loveridgei* – IBSP 10126

*A. maculatus* – IBSP 40003, IBSP 55213

*A. major* – USNM 232650, IBSP 43395

*A. oculotemporalis* – IBSP 10227

*A. panctostictus* – IBSP 54655

*A. reticulatus* – IBSP 48815, IBSP 48853

*A. resplendens* – AMNH 35932

*A. schach* – IBSP 43396

*A. serranus* – IBSP 54636

*A. snethlageae* – IBSP 46454

*A. trilineatus* – IBSP 25783

*A. triredrurus* – IBSP 9088

*Chersodromus liebmanni* – USNM 109932

*Coniophanes* *imperalis* – KU 70828

*C. fissidens* – KU 190902

*C. bipunctatus* – KU 203168

*C. quinquevittatus* – AMNH 93119

*C. piceivittis* – KU 35554

*Crisantophis nevermanni* – USMN 194378, KU 174470, LSUMZ 35309

*Cryophis hallbergi* – KU 200973

*Enulius* *flavitorques* – KU 112614, KU 174188

*E. unicolor* – AMNH 8002

*Enuliophis* *sclateri* – KU 112617, KU 34047

*Geophis anocularis* – KU 200975

*G.* *brachycephalus* – KU 63802, KU 63805

*G. dubius* – AMNH 102954

*G. hoffmanni* – AMNH 113561

*G. multitorques* – LSUMZ 11015

*G. rhondogaster* – KU 187272

*G. zeledoni* – KU 63822, KU 103884

*Hypsiglena torquata* – KU 192276, KU 193259, KU 73522

*Imantodes* *cenchoa* – MPEG 15318

*I.* *inornatus –* KU 174207

*I. gemmistratus* – KU 174207

*I. lentiferus* – KU 121899, KU 112270

*Leptodeira annulata* – IBSP 62490, IBSP 62102, KU 183896

*L. frenata* – MZUSP 6437

*L. maculata* – KU 62490, KU 62480

*L. nigrofasciata* – KU 174247

*L. punctata* – KU 75623

*L. splendida* – KU 73553

*L. septentrionalis* – KU 190983, KU 187324, MZUSP 6435

*Ninia* *atrata* – AMNH 59426, KU 63850, KU 35528, USNM 228096

*N. diademata* – AMNH 118002

*N. maculata* – KU 30932, KU 30943

*N. psephota* – KU 331972

*N. sebae* – KU 209342, KU 84879. KU 84877

*Pliocercus elapoides* – AMNH 102981

*P. eryzonus* – USNM 150136

*Rhadinaea flavilata –* AMNH 63891

*R. godmani* – KU 194346

*R. montecristi* – KU 63881

*Tretanorhinus* *variabilis* – KU 268974, KU 174409, KU 268973

*Urotheca* *decipiens* – KU 103893, KU 112455
