# Supplementary figures and images for: Consuming viscous prey: a novel protein-secreting delivery system in neotropical snail-eating snakes
Source: BMC Evol Biol. 2014 Mar 25;14:58. doi: 10.1186/1471-2148-14-58 (PMC4021269; doi:10.1186/1471-2148-14-58)

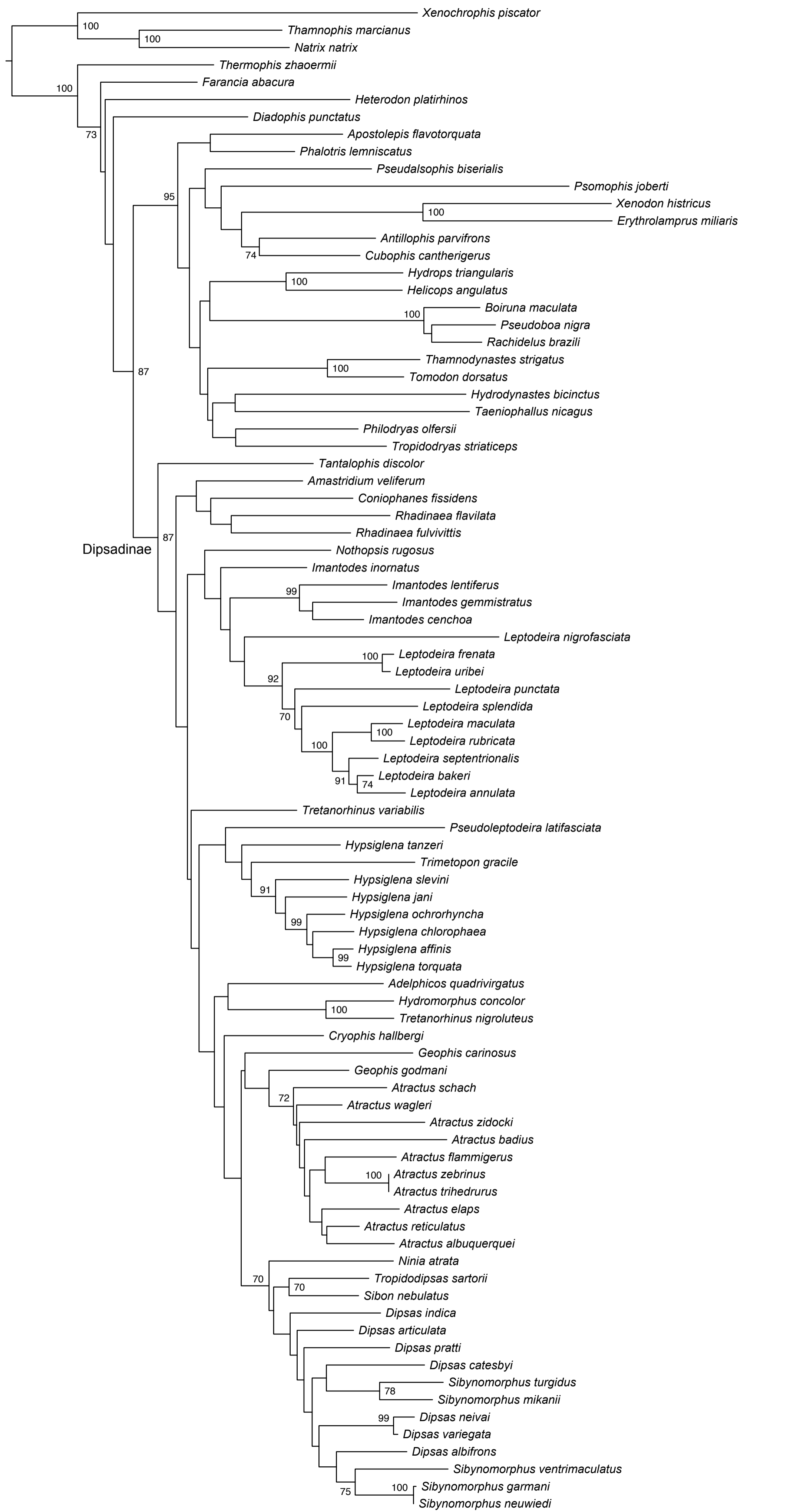

Supplement: Additional file 3 — Tree estimated from a Maximum Likelihood analysis of 11 concatenated genes using RAxML 7.2.8. All outgroups are shown in the tree. Bootstrap values greater than 70% are given above each node. [file 1471-2148-14-58-S3.pdf]

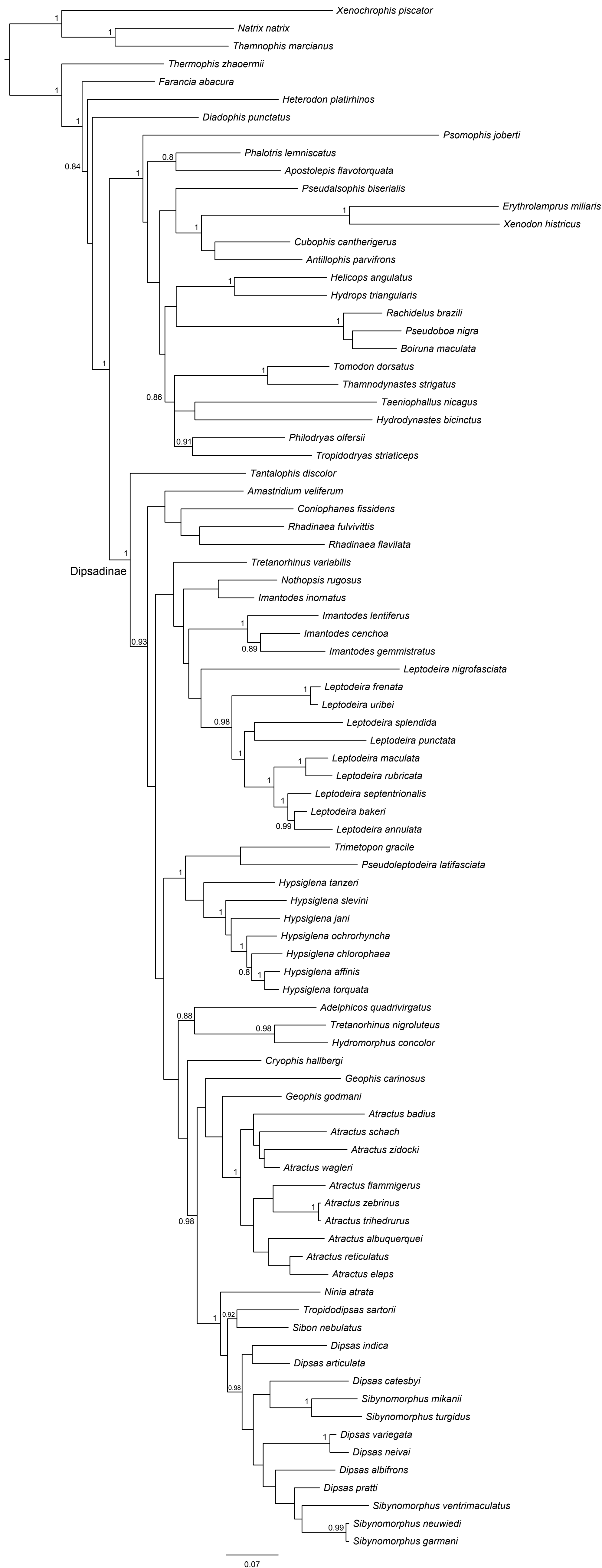

Supplement: Additional file 4 — Fifty percent Majority-rule consensus tree estimated from a Bayesian analysis of 11 concatenated genes using MrBayes 3.1.2. All outgroups are shown in the tree. Bayesian Posterior Probability frequencies greater than 0.80 are given above each node. [file 1471-2148-14-58-S4.pdf]
