# Supplementary material for: Consuming viscous prey: a novel protein-secreting delivery system in neotropical snail-eating snakes
Source: BMC Evol Biol. 2014 Mar 25;14:58. doi: 10.1186/1471-2148-14-58 (PMC4021269; doi:10.1186/1471-2148-14-58)
Supplement: Additional file 6 — A List of DNA sequences used in this study, with GenBank accession numbers. [file 1471-2148-14-58-S6.doc]

BMC Evolutionary Biology

**Additional File 6**

**Article: Consuming viscous prey: A Novel Protein-Secreting Delivery System in Neotropical Snail-Eating Snakes**

Authors: Hussam Zaher, Leonardo de Oliveira, Felipe G Grazziotin, Michelle Campagner, Carlos Jared, Marta M Antoniazzi, Ana L Prudente

List of sequences used in this study. All accession numbers listed below are from GenBank.

| **Taxa** | **12S** | **16S** | **cytb** | **nd2** | **nd4** | **bdnf** | **cmos** | **jun** | **nt3** | **rag1** | **rag2** |
| --- | --- | --- | --- | --- | --- | --- | --- | --- | --- | --- | --- |
| *Adelphicos quadrivirgatus* | - | - | GQ895853 | - | - | - | GQ895796 | - | - | - | - |
| *Amastridium veliferum* | - | - | GQ334479 | - | GQ334580 | - | GQ895797 | - | GQ334663 | - | - |
| *Antillophis parvifrons* | YPX108 | YPX108 | FJ416740  YPX108 | FJ416778 | FJ416814 | JQ599006 | - | YPX108 | YPX108 | YPX108 | FJ416851 |
| *Apostolepis flavotorquata* | APFL001-ZZ | JQ598858 | GQ895854 | - | - | - | JQ598967 | - | - | - | - |
| *Atractus albuquerquei* | GQ457783 | GQ457726 | JQ598918 | - | - | JQ599009 | GQ457845 | YPX110 | - | - | - |
| *Atractus badius* | AF158425 | AF158485 | - | - | - | - | - | - | - | - | - |
| *Atractus elaps* | - | - | EF078536 | - | EF078584 | - | - | - | - | - | - |
| *Atractus flammigerus* | AF158402 | AF158471 | - | - | - | - | - | - | - | - | - |
| *Atractus reticulatus* | JQ598798 | - | - | - | - | - | JQ598970 | - | - | - | - |
| *Atractus schach* | JQ598799 | JQ598860 | - | - | - | - | JQ598971 | - | - | - | - |
| *Atractus trihedrurus* | GQ457784 | GQ457727 | JQ598919 | - | - | JQ599010 | GQ457846 | YPX112 | YPX112 | YPX112 | - |
| *Atractus wagleri* | - | - | GQ334480 | - | GQ334581 | - | - | - | GQ334664 | - | - |
| *Atractus zebrinus* | JQ598800 | JQ598861 | - | - | - | - | JQ598972 | - | - | - | - |
| *Atractus zidocki* | AF158426 | AF158487 | - | - | - | - | - | - | - | - | - |
| *Boiruna maculata* | GQ457785 | JQ598862 | JQ598920 | - | - | JQ599011 | GQ457847 | - | YPX113 | YPX113 | - |
| *Coniophanes fissidens* | - | - | EF078538 | - | EF078586 | - | - | - | - | - | - |
| *Cryophis hallbergi* | - | - | EF078496 | - | GQ334582 | - | GQ895807 | - | GQ334666 | - | - |
| *Cubophis cantherigerus* | AF158405 | AF158475 | AF544669 | FJ416782 | FJ416818 | FJ433999 | AF544694 | EF144057 | FJ434100 | AY487376 | EF144109 |
| *Diadophis punctatus* | AY577015 | YPX089 | EU193843 | - | EU194025 | JQ599017 | AF471122  AF544705  YPX089 | YPX089 | YPX089 | AY487403 | EF144110 |
| *Dipsas albifrons* | JQ598803 | JQ598866 | - | - | - | JQ599019 | - | - | YPX117 | - | - |
| *Dipsas articulata* | JQ598804 | JQ598867 | - | - | - | JQ599020 | - | YPX118 | YPX118 | YPX118 | - |
| *Dipsas catesbyi* | JQ598805 | JQ598868 | JQ598926 | - | EF078585 | JQ599021 | JQ598977 | YPX119 | YPX119 | YPX119 | - |
| *Dipsas indica* | GQ457789 | GQ457730 | - | - | - | - | GQ457850 | - | - | - | - |
| *Dipsas neivai* | GQ457790 | GQ457731 | - | - | - | - | GQ457851 | - | - | - | - |
| *Dipsas pratti* | - | - | GQ334482 | - | GQ334583 | - | - | - | GQ334667 | - | - |
| *Dipsas variegata* | AF158406 | AF158476 | - | - | - | - | - | - | - | - | - |
| *Erythrolamprus miliaris* | JQ598811 | JQ598874 | JQ598931 | - | - | JQ599025 | JQ598982 | YPX129 | YPX129 | YPX129 | - |
| *Farancia abacura* | Z46467 | Z46491 | U69832 | DQ902239 | DQ902307 | - | AF471141 | - | - | - | - |
| *Geophis carinosus* | - | - | GQ895872 | - | - | - | GQ895815 | - | - | - | - |
| *Geophis godmani* | JQ598814 | JQ598877 | JQ598932 | - | - | JQ599026 | - | - | YPX123 | - | - |
| *Helicops angulatus* | GQ457797 | GQ457738 | AF471037 | FJ416751 | - | - | AF471160 | - | - | - | FJ416824 |
| *Heterodon platirhinos* | AY577019 | YPX568 | JQ598934 | FJ416750 | AF402659 | JQ599028 | JQ598986 | - | - | YPX568 | FJ416823 |
| *Hydrodynastes bicinctus* | GQ457802 | GQ457742 | JQ598935 | - | - | JQ599030 | GQ457862 | - | YPX125 | YPX125 | - |
| *Hydromorphus concolor* | - | - | GQ895874 | - | - | - | GQ895817 | - | - | - | - |
| *Hydrops triangularis* | GQ457804 | GQ457744 | AF471039 | - | - | JQ599032 | GQ457864  AF471158 | - | YPX126 | - | - |
| *Hypsiglena affinis* | - | - | - | - | EU363055 | - | - | - | - | - | - |
| *Hypsiglena chlorophaea* | EU728577 | EU728577 | EU728593 | EU728585 | NC13977 | - | - | - | FJ455197 | - | - |
| *Hypsiglena jani* | EU728592 | EU728592 | EU728592 | NC13975 | EF078551 | - | - | - | FJ455193 | - | - |
| *Hypsiglena ochrorhyncha* | EU728578 | EU728578 | EU728578 | EU728578 | EU728578 | - | - | - | FJ455199 | - | - |
| *Hypsiglena slevini* | EU728584 | EU728584 | EU728584 | NC13987 | EU728584 | - | - | - | FJ455191 | - | - |
| *Hypsiglena tanzeri* | - | - | EU728588 | - | EU363044 | - | - | - | - | - | - |
| *Hypsiglena torquata* | EU728591 | EU728591 | EU728591 | EU728591 | NC13992 | - | AF471159 | - | FJ455192 | - | - |
| *Imantodes cenchoa* | EU728586  YPX127 | EU728586  YPX127 | EU728586  YPX127 | NC13988 | NC13988 | JQ599033 | GQ457865 | - | YPX127 | YPX127 | - |
| *Imantodes gemmistratus* | - | - | EF078509 | - | GQ334588 | - | - | - | - | - | - |
| *Imantodes inornatus* | - | - | EF078511 | - | EF078559 | - | - | - | - | - | - |
| *Imantodes lentiferus* | AF158463 | AF158532 | EF078514 | - | EF078561 | - | - | - | - | - | - |
| *Leptodeira annulata* | GQ457806 | GQ457746 | EF078516 | FJ416749 | FJ416787 | FJ433998 | AF544690  GQ457866 | EF144056 | FJ434099 | AY487375 | EF144108 |
| *Leptodeira bakeri* | - | - | GQ334518 | - | GQ334622 | - | - | - | GQ334673 | - | - |
| *Leptodeira frenata* | - | - | EF078532 | - | FJ810244 | - | - | - | - | - | - |
| *Leptodeira maculata* | - | - | GQ334521 | - | GQ334626 | - | - | - | GQ334674 | - | - |
| *Leptodeira nigrofasciata* | - | - | EF078533 | - | GQ334629 | - | - | - | GQ334681 | - | - |
| *Leptodeira punctata* | - | - | EF078530 | - | EF078577 | - | - | - | GQ334682 | - | - |
| *Leptodeira rubricata* | - | - | GQ334527 | - | GQ334631 | - | - | - | - | - | - |
| *Leptodeira septentrionalis* | EU728590 | EU728590 | EF078527 | NC13990 | GQ334636 | - | - | - | FJ455188 | - | - |
| *Leptodeira splendida* | - | - | EF078521 | - | EF078569 | - | - | - | GQ334680 | - | - |
| *Leptodeira uribei* | - | - | FJ810235 | - | - | - | - | - | - | - | - |
| *Natrix natrix* | YPX538 | EU078986 | AY866541  YPX538 | AY870624 | AY873716 | JQ599036 | AF471121  AF544697  YPX539 | YPX538 | - | EU402858 | - |
| *Ninia atrata* | GQ457814 | JQ598882 | JQ598937 | - | GQ334659 | JQ599037 | GQ457874 | YPX131 | YPX131 | YPX131 | - |
| *Nothopsis rugosus* | GU018159 | GU018177 | - | - | - | - | - | - | - | - | - |
| *Phalotris lemniscatus* | GQ457817 | GQ457756 | - | - | - | JQ599039 | GQ457877 | - | - | - | - |
| *Philodryas olfersii* | JQ598830 | JQ598890 | JQ598945 | - | - | JQ599041 | JQ598993 | - | YPX134 | YPX134 | - |
| *Pseudalsophis biserialis* | JQ598832 | JQ598892 | JQ598946 | - | - | - | JQ598994 | YPX107 | YPX107 | YPX107 | - |
| *Pseudoboa nigra* | YPX136 | GQ457764 | JQ598948 | - | - | JQ599043 | GQ457885  AF544729 | YPX136 | YPX136 | - | - |
| *Pseudoleptodeira latifasciata* | EU728579 | EU728579 | EU728579 | NC13981 | NC13981 | - | - | - | FJ455190 | - | - |
| *Psomophis joberti* | GQ457829 | GQ457768 | JQ598950 | - | - | JQ599046 | GQ457889 | YPX137 | YPX137 | YPX137 | - |
| *Rhachidelus brazili* | JQ598837 | JQ598897 | JQ598952 | - | - | JQ599048 | - | YPX139 | YPX139 | YPX139 | - |
| *Rhadinaea flavilata* | - | - | AF471078 | - | - | - | AF471152 | - | - | - | - |
| *Rhadinaea fulvivittis* | - | - | EF078539 | - | EF078587 | - | - | - | - | - | - |
| *Sibon nebulatus* | EU728583 | EU728583 | EU728583 | NC13985 | NC13985 | - | AF544736 | - | FJ455189 | - | - |
| *Sibon sartorrii* | - | - | EF078540 | - | EF078588 | - | - | - | - | - | - |
| *Sibynomorphus garmani* | GQ457831 | GQ457770 | - | - | - | - | GQ457891 | - | - | - | - |
| *Sibynomorphus mikanii* | GQ457832 | GQ457771 | JQ598954 | - | - | JQ599050 | GQ457892 | YPX141 | YPX141 | YPX141 | - |
| *Sibynomorphus neuwiedii* | JQ598838 | JQ598898 | - | - | - | - | - | - | - | - | - |
| *Sibynomorphus turgidus* | JQ598839 | JQ598899 | - | - | - | - | - | - | - | - | - |
| *Sibynomorphus ventrimaculatus* | JQ598840 | JQ598900 | - | - | - | - | JQ598997 | - | - | - | - |
| *Taeniophallus nicagus* | JQ598845 | JQ598906 | YPX146 | - | - | YPX146 | - | - | YPX146 | YPX146 | - |
| *Tantalophis discolor* | - | - | EF078541 | - | EF078589 | - | - | - | - | - | - |
| *Thamnodynastes strigatus* | JQ598847 | JQ598907 | JQ598959 | - | - | JQ599057 | - | YPX147 | YPX147 | YPX147 | - |
| *Thamnophis marcianus* | AF402643 | - | AF420143 | AF384845 | AF420146 | EU402660 | - | - | - | EU402862 | - |
| *Thermophis zhaoermii* | NC012816 | GQ166168 | GQ166168 | NC012816 | GQ166168 | - | NC012816 | - | - | - | - |
| *Tomodon dorsatus* | GQ457838 | GQ457777 | JQ598960 | - | - | JQ599059 | GQ457897 | YPX148 | YPX148 | YPX148 | - |
| *Tretanorhinus nigroluteus* | - | - | GQ895893 | - | - | - | GQ895834 | - | - | - | - |
| *Tretanorhinus variabilis* | AF158460 | AF158529 | - | - | - | - | - | - | - | - | - |
| *Trimetopon gracile* | GU018160 | GU018178 | - | - | - | - | - | - | - | - | - |
| *Tropidodryas striaticeps* | GQ457839 | GQ457778 | YPX149 | - | - | JQ599060 | - | YPX149 | YPX149 | - | - |
| *Xenochrophis piscator* | YPX546 | YPX546 | GQ225659 | - | - | YPX546 | YPX546 | - | YPX546 | EU402868 | - |
| *Xenodon histricus* | GQ457813 | GQ457753 | JQ598962 | - | - | JQ599061 | GQ457873 | YPX130 | - | YPX130 | - |
